# Circadian Profiling of the Arabidopsis Proteome Using 2D-DIGE

**DOI:** 10.3389/fpls.2016.01007

**Published:** 2016-07-12

**Authors:** Mani K. Choudhary, Yuko Nomura, Hua Shi, Hirofumi Nakagami, David E. Somers

**Affiliations:** ^1^Division of Integrative Biosciences and Biotechnology, Pohang University of Science and TechnologyPohang, South Korea; ^2^Plant Proteomics Research Unit, RIKEN Center for Sustainable Resource ScienceYokohama, Japan; ^3^Department of Molecular Genetics, Ohio State UniversityColumbus, OH, USA

**Keywords:** circadian clock, proteomics, abscisic acid (ABA) signaling, chaperones, 2D-DIGE

## Abstract

Clock-generated biological rhythms provide an adaptive advantage to an organism, resulting in increased fitness and survival. To better elucidate the plant response to the circadian system, we surveyed protein oscillations in Arabidopsis seedlings under constant light. Using large-scale two-dimensional difference in gel electrophoresis (2D-DIGE) the abundance of more than 1000 proteins spots was reproducibly resolved quantified and profiled across a circadian time series. A comparison between phenol-extracted samples and RuBisCO-depleted extracts identified 71 and 40 rhythmically-expressed proteins, respectively, and between 30 and 40% of these derive from non-rhythmic transcripts. These included proteins influencing transcriptional regulation, translation, metabolism, photosynthesis, protein chaperones, and stress-mediated responses. The phasing of maximum expression for the cyclic proteins was similar for both datasets, with a nearly even distribution of peak phases across the time series. STRING clustering analysis identified two interaction networks with a notable number of oscillating proteins: plastid-based and cytosolic chaperones and 10 proteins involved in photosynthesis. The oscillation of the ABA receptor, PYR1/RCAR11, with peak expression near dusk adds to a growing body of evidence that intimately ties ABA signaling to the circadian system. Taken together, this study provides new insights into the importance of post-transcriptional circadian control of plant physiology and metabolism.

## Introduction

The circadian clock controls many aspects of plant metabolism and development and is most closely associated with the transcriptional regulation of these processes. Numerous genetic studies in plants have examined the performance of photosynthesis, growth, and survival under stresses in clock mutant backgrounds, and changes in the gene expression of key elements of these processes are typically found (Green et al., 2002; Dodd et al., 2005; Mizuno and Yamashino, 2008; Fukushima et al., 2009; Legnaioli et al., 2009; Graf et al., 2010; Greenham and McClung, 2015). Transcriptome analyses have implicated the circadian clock in the rhythmic control of mRNA levels (Harmer et al., 2001; Covington et al., 2008; Hazen et al., 2009; Filichkin et al., 2011; Nose and Watanabe, 2014) and genome-wide studies have shown the wide-ranging roles of clock components in the control of gene expression (Gendron et al., 2012; Huang et al., 2012; Nakamichi et al., 2012; Hsu et al., 2013; Liu et al., 2013; Nagel et al., 2015). These findings include clock control of the plant circadian oscillator itself as well as many output genes involved in metabolism, physiology, and development.

Fewer studies, both in plants and animals, have examined the circadian control of post-transcriptional processes such as mRNA and protein turnover, and post-translational modifications such as phosphorylation, ubiquitinylation, and sumoylation (Kim et al., 2003; Cardone et al., 2005; Reddy et al., 2006; Deery et al., 2009; Mehra et al., 2009; Mauvoisin et al., 2014a,b; Seo and Mas, 2014; van Wijk et al., 2014; Choudhary et al., 2015; Nolte and Staiger, 2015). Additionally, the presumption that protein oscillations will follow from transcript cycling is not always borne out. About 20–50% of circadian cycling proteins in the liver lack accompanying mRNA oscillations (Reddy et al., 2006; Mauvoisin et al., 2014b; Robles et al., 2014) and in the suprachiasmatic nucleus only between 11 and 38% of the rhythmic proteins show significantly rhythmic mRNA (Deery et al., 2009). Studies like these indicate that transcriptome kinetics do not necessarily predict proteomic profiles.

Therefore, analysis of the proteome is essential to understand how cellular processes respond to, and are controlled by, the circadian clock. Since the development of proteomic techniques in the last few decades rhythmic proteins have been detected using high throughput methods including two-dimensional in gel electrophoresis (2-DE), mass spectrometry (MS), and MALDI-TOF. Proteomic approaches have been used to identify clock-associated proteins and circadian oscillations in proteins in dinoflagellates (Akimoto et al., 2004), *Chlamydomonas reinhardtii* (Wagner et al., 2004; Wagner and Mittag, 2009), and *Ostreococcus taurii* (Le et al., 2011). Analysis of the clock-regulated proteome of higher plants has been limited to one study in rice (Hwang et al., 2011) and the Arabidopsis phosphoproteome (Choudhary et al., 2015).

To further study the changes in Arabidopsis circadian proteome and to deepen our understanding of the extent of clock control of protein oscillations, we use two-dimensional difference in gel electrophoresis (2D-DIGE) to identify polypeptides in Arabidopsis seedlings that oscillate in abundance under constant light. Through the inclusion of an internal standard (IS) for normalization, this technique allows the analysis of up to three pools of protein samples simultaneously on a single 2D gel, thereby minimizing gel-to-gel variability (Alban et al., 2003). We utilized two different methods of sample preparation. A comprehensive total protein extract (phenol extraction) was compared to the protein profile obtained from D-ribulose bisphosphate carboxylase/oxygenase (RuBisCO)-depleted samples. This immuno-affinity approach was applied in an effort to enhance the detection of low abundance proteins that might be masked by the very high levels of RuBisCO present in green tissue (Sehrawat et al., 2013; Aryal et al., 2015).

Together more than 100 oscillating proteins were identified that are involved in transcriptional regulation, translation, metabolism, photosynthesis, protein chaperones, and stress-mediated responses. We were able to highlight previously undescribed protein oscillations in chaperones involved in chloroplast import and photosynthesis and make new connections between the circadian clock and ABA signaling.

## Materials and methods

### Plant materials and growth conditions

Arabidopsis Columbia ecotype seeds were surface sterilized and cold-treated for 2 days to synchronize germination. Seeds were plated on filter paper in petri dish containing growth medium Murashige and Skoog basal salt mixture, 3.0% sucrose, and 1.0% agar (pH 5.7). Seedlings were grown under 12 h white fluorescent light (120 μmol m^−2^ s^−1^), 12 h light/12 h dark cycle for 10 days at 22⋅C and then maintained under constant light for 24 h before harvesting. The tissues were harvested at the indicated time points (LL25, LL29, LL 33, LL37, LL41, and LL45) and frozen immediately (Table S1).

### Extraction of total proteins for 2D-dige

Total proteins were extracted with the phenol–methanol method (Hurkman and Tanaka, 1986) with modifications. Approximately 0.1 g of tissue were ground into fine powder in liquid nitrogen and mixed with three volumes of SDS extraction buffer (100 mM Tris-HCl, pH 8.0, 2% SDS, 1% β-mercaptoethanol, 5 mM EGTA, 10 mM EDTA), vortexed vigorously, and centrifuged at 20,000 × g for 20 min. The supernatant was mixed with an equal volume of ice-cold phenol (Tris-buffered, pH 7.5–7.9) and centrifuged at 20,000 × g for 15 min at 4⋅C to separate phenol and aqueous phases. The upper aqueous phase was removed leaving the interface intact, and the phenol phase was extracted twice with 50 mM Tris-HCl, pH 8.0. It was then mixed with five volumes of cold 0.1 M ammonium acetate in methanol and held at −20⋅C overnight to precipitate proteins. After centrifugation at 20,000 × g for 20 min, the protein pellet was washed three times with 1 ml of cold 0.1 M ammonium acetate in methanol and once with ethanol and then resuspended in buffer (30 mM Tris-Cl, 7 M urea, 2 M thiourea, 4% CHAPS pH 8.5). After centrifugation, the supernatant was transferred to a new tube, and the solubilized protein sample concentration was quantified with 2-D quant kit (GE Healthcare) using BSA as a standard.

### RuBisCO depletion by immunoaffinity purification

Seedlings were ground into fine powder in liquid nitrogen and homogenized in (1:3, w/v) buffer (10 mM Tris pH 7.5, 150 mM NaCl, 0.5% NP-40, protease inhibitor). The homogenate was centrifuged at 12,000 rpm for 20 min at 4⋅C. The protein concentration was determined with the Bio-Rad protein assay using BSA as a standard. The supernatant was subjected to immunoaffinity purification (Seppro IgY RuBisCO Spin Column kit, Sigma-Aldrich) following the manufacturer's instructions. Briefly, the column was pre-washed thrice with 500 μL Tris buffered saline (TBS, 1 mM Tris-HCl, 150 mM NaCl, and pH 7.4). Immuno-capture of RuBisCO was performed by incubating the supernatant (100 μg protein) with the matrix for 15 min at 25⋅C with gentle shaking. After 15 min, the flow through was collected by centrifugation at 2000 rpm for 30 s. Unbound protein were removed by washing with TBS. Elution was done with the stripping buffer (100 mM glycine-HCl, pH 2.5) and the fractions were immediately neutralized with 1 M Tris-HCl, pH 8.0.

### Protein CyDye labeling

Proteins were labeled with DIGE-specific Cy2, Cy3, or Cy5 according to the manufacturer's instructions (GE Healthcare) with modifications. Briefly, after adjusting pH to 8.5 using NaOH (100 mM), 50 μg of proteins were mixed with 400 pmol of CyDye and incubated on ice in the dark for at least 10 min. The reaction was stopped by addition of 1.0 μl of 10 mM lysine and incubated on ice for 10 min. Each sample was covalently labeled with a fluorophore, either Cy3 or Cy5. A mixture of equal amounts of protein from every sample in the experiment was labeled with Cy2 and used as internal standard.

### Two-dimensional gel electrophoresis

For analytical 2D-DIGE analysis 50 μg each of Cy3-, Cy5-, and Cy2-labeled protein samples were mixed together (total 150 μg of protein). The DIGE sample buffer [7 M urea, 2 M thiourea, 4% CHAPS, 20 mM DTT, and 0.5% IPG buffer (GE Healthcare)] was added to bring the volume to 450 μl, and the samples were then applied to 24-cm Immobiline Drystrips (GE Healthcare) and rehydrated overnight. IEF was carried out on an Ettan IPGphor II (GE Healthcare) at 20⋅C with a maximum of 50 μA/strip and the following setting: 500 and 1000 V each for 1 h, a gradient increase to 8000 V over 3 h, and remaining at 8000 V until an accumulated voltage of reaching the desired total V-h (72,000 for pH 4–7 IPG strips). After IEF, IPG strips were equilibrated in equilibration buffer [6 M urea, 30% (w/v) glycerol, 2% SDS, 50 mM Tris-HCl, pH 8.0] first with 0.5% DTT and then with 2% iodoacetamide each for 15 min. The equilibrated strips were then transferred to 12.5% SDS-PAGE gels for the second dimension electrophoresis using the Ettan Dalt-six (GE Healthcare/Amersham Biosciences) vertical unit. SDS-PAGE was run overnight: the first step at 80 V, for 1 h, the second step at 120 V, until the bromophenol blue dye front reached the bottom of gel. Four biological trials were performed at each time point.

### 2D-DIGE image analysis

Gels were processed using a Typhoon 9410 scanner (GE Healthcare/Amersham Biosciences) according to the manufacturer's recommendations for DIGE image analysis. The DIGE images were analyzed using Progenesis Same Spots software v4.0 (Nonlinear Dynamics, Durham, NC). The scanned images were matched, and artifacts, damaged areas, and spots on the gels or on the scanner bed were removed. Analysis using Progenesis software included spot detection, background subtraction, normalization, and matching. Normalization was done using an internal standard (IS) sample. The internal pooled standard allows the comparison of more than two proteomes without the need to perform pair-wise analysis of all possible combinations of data points. After automatic spot detection, manual editing was performed to ensure that spots were correctly matched between different gels and were not contaminated with artifacts, such as streaks or dust. Spots were manually revised with edition tools for correct detection. Gel groups were established according to the experimental design and spot normalized volume was used to select statistically significant (fold change, ANOVA) differentiated spots between time points. The setting was fixed in each experiment so that fewer than three spots presence on at least three of the four replicates, including presence on the reverse labeled one would be considered as a false positive.

Preparative 2D gels loaded with 1 mg of protein were used for spot picking. These gels were loaded with equal amounts of pooled samples from all 24 samples (six time points from four biological trials). After electrophoresis the gel was silver stained, scanned with a transmission-light densitometer (Image Scanner; GE Healthcare), and aligned with the DIGE reference image with Progenesis SameSpots to outline the spots of interest selected in the previous analysis. The spots were excised manually from three preparative gels for each extraction method.

### In-gel tryptic digestion, mass spectrometry, and database searching

In-gel digestions were performed as described previously (Shevchenko et al., 2006). Digested peptides in the gel pieces were recovered by adding 5% formic acid/acetonitrile, desalted using StageTips with C18 disk membranes (EMPORE, 3M; Rappsilber et al., 2003), dried in a vacuum evaporator, and dissolved in 9 μL of 5% acetonitrile containing 0.1% trifluoroacetic acid. An LTQ-Orbitrap XL (Thermo Fisher Scientific) coupled with an EASY-nLC 1000 (Thermo Fisher Scientific) was used for nano-LC-MS/MS analyses. A self-pulled needle (150 mm length × 100-μm i.d., 6-μm opening) packed with ReproSil C18 resin (3 μm; Dr. Maisch GmbH) was used as an analytical column with “stone-arch” frit (Ishihama et al., 2002). A spray voltage of 2400 V was applied. The injection volume was 6 μL, and the flow rate was 500 nL min^−1^. The mobile phase consisted of 0.5% acetic acid (A) and 0.5% acetic acid and 80% acetonitrile (B). A two-step linear gradient of 0–40% B in 30 min, 40–100% B in 5 min, and 100% B for 10 min was employed. The MS scan range was m/z 300–1400. The top 10 precursor ions were selected in the MS scan by Orbitrap at 100,000 resolution and for subsequent MS/MS scans by ion trap in the automated gain control mode, where automated gain control values of 5.00e + 05 and 1.00e + 04 were set for full MS and MS/MS, respectively. The normalized collision-induced dissociation was set to 35.0. A lock mass function was used for the LTQ-Orbitrap XL to obtain constant mass accuracy during gradient analysis (Olsen et al., 2005). Selected sequenced ions were dynamically excluded for 60 s after sequencing. Mass Navigator version 1.3 (Mitsui Knowledge Industry, Tokyo, Japan) with default parameters for LTQ-Orbitrap XL was used to create peak lists on the basis of the recorded fragmentation spectra. The m/z-values of the isotope peaks were converted to the corresponding monoisotopic peaks when the isotope peaks were selected as the precursor ions. To improve the quality of the MS/MS spectra, Mass Navigator discarded all peaks of < 10 absolute intensity and with < 0.1% of the most intense peak in MS/MS spectra (Ravichandran et al., 2009). Peptides and proteins were identified by means of automated database searching using Mascot version 2.3.02 (Matrix Science) in The Arabidopsis Information Resource database (TAIR10_pep_20101214, ftp://ftp.arabidopsis.org/home/tair/Sequences/blast_datasets/TAIR10_blastsets/) with a precursor mass tolerance of 3 ppm, a fragment ion mass tolerance of 0.8 Da, and strict trypsin specificity (Olsen et al., 2004), allowing for up to two missed cleavages. Carbamidomethylation of Cys was set as a fixed modification, and oxidation of Met and phosphorylation of Ser, Thr, and Tyr were allowed as variable modifications. Scaffold (version Scaffold_4.5.3, Proteome Software Inc., Portland, OR) was used to validate MS/MS based peptide and protein identifications. Peptide identifications were accepted if they could be established at >95.0% probability by the Peptide Prophet algorithm (Keller et al., 2002). Protein identifications were accepted if they could be established at >99.9% probability and contained at least two identified peptides. Protein probabilities were assigned by the Protein Prophet algorithm (Nesvizhskii et al., 2003). Proteins that contained similar peptides and could not be differentiated based on MS/MS analysis alone were grouped to satisfy the principles of parsimony. Contaminants were removed manually. The mass spectrometry proteomics data have been deposited to the ProteomeXchange Consortium via the PRIDE partner repository with the dataset identifier PXD004276 (Vizcaíno et al., 2016).

### Immunoblotting

Growth conditions and harvest of Arabidopsis seedlings were as described previously (Choudhary et al., 2015). Total protein extraction was performed according to Fujiwara et al. (2008). Protein extracts were size-fractionated by 12% SDS-PAGE. Immunoblotting was performed using polyclonal RuBisCO activase antibody (aA-18, sc-15864, Santa Cruz Biotechnology) with 1:3000 dilution. RCA is known to be phosphorylated and has two detectable isoforms in Arabidopsis, 43 and 47 kDa (Yin et al., 2010). Hence, all detectable bands were used for quantitation. Histone H3 antibody (ab1791, Abcam) was used for loading control with 1:2000 dilution. Image J software was used for quantification of protein signal intensity.

### GO, STRING, and SOTA analysis

For Gene Ontology (GO) enrichment information for differentially expressed significant proteins data sets, the three GO vocabularies, biological processes, cellular component, and molecular function were searched using the GO Slim Classification for plants developed at TAIR (https://www.arabidopsis.org/tools/bulk/go/index.jsp). The AGI accession numbers for Arabidopsis were uploaded and ontology of GO cellular component, biological process, and molecular function was chosen with other settings as default.

A functional network of circadian modulated proteins was predicted using STRING version 10.0 (Szklarczyk et al., 2015; available at www.string-db.org). Functional protein-association networks were visualized with high confidence (0.7), with *Arabidopsis thaliana* set as the organism.

Self-organizing tree algorithm (SOTA) clustering was used to obtain the co-expression pattern of differentially expressed significant proteins (Herrero et al., 2001). Clustering was performed on log-transformed fold induction expression values across six time points using Multi Experiment Viewer (MEV) software (The Institute for Genomic Research). The Pearson correlation distance was set at 10 cycles and a maximum cell diversity of 0.9 (Romijn et al., 2005).

### RNA extraction and quantitative RT-PCR

Total RNA was extracted with RNAzol reagents (Sigma) according to the manufacturer's instructions from Col-0 seedlings grown on MS media. After digestion with DNase I (Invitrogen), 2 μg of total RNA was used to synthesize cDNA by using oligo-dT and SuperScriptIII reverse transcriptase (Invitrogen) following the manufacturer's instructions. The *PYR1* gene-specific primers were PYR1qFP: TCCTGCTCCGTCGAACAAAACTTC and PYR1qRP: CGCCTC CGATGATACTGAATCCG. The *UBQ* primers were UBQ qFP: TGCGCTGCCAGATAA TACACTATT and UBQ qRP: TGCTGCCCAACATCAGGTT. qRT-PCR was performed using iQ SYBR Green Supermix (Bio-Rad) and Bio-Rad CFX96 real-time PCR detection system.

## Results and discussion

### 2D-DIGE reveals circadian control over protein abundance patterns

To gain insight into the circadian regulation of protein expression patterns, we analyzed the proteome of Arabidopsis seedlings after entrainment in 12 h light and 12 h dark cycles followed by free run under constant white light for 24 h. Samples were subsequently collected at 4 h intervals starting at LL25. Quantitative proteomics analysis was performed using two-dimensional difference in gel electrophoresis (2D-DIGE). To obtain optimal separation of Arabidopsis proteins, we first used a phenol-methanol extraction method (Hurkman and Tanaka, 1986). In a second approach, RuBisCO-depleted samples were tested in an effort to enhance the detection of low abundance proteins that might be masked the very high levels of RuBisCO present in green tissue (Sehrawat et al., 2013; Aryal et al., 2015).

**Figure 1:**
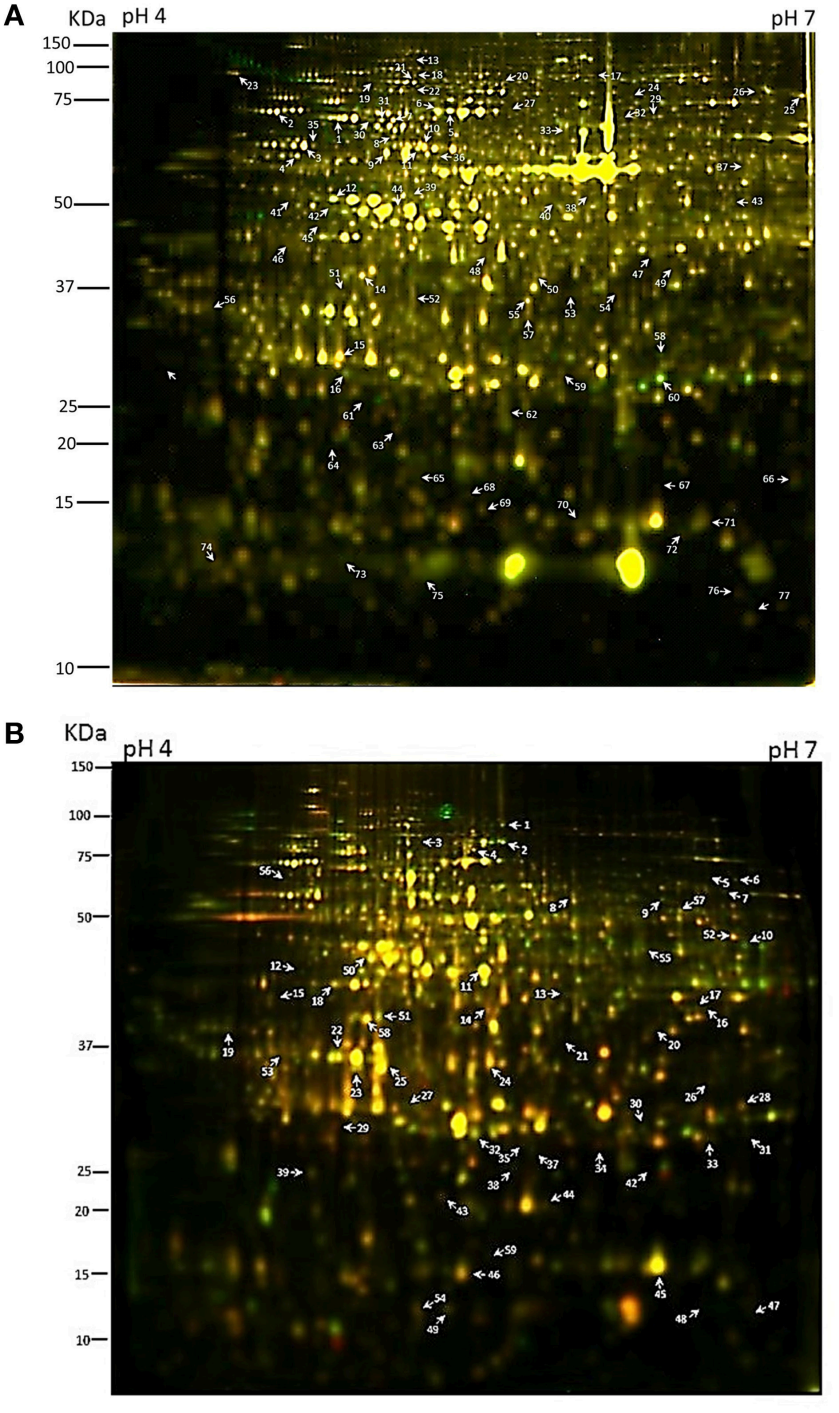
**Two-dimensional difference gel electrophoresis (2-D DIGE) analysis of circadian regulated proteins. (A)** Total protein isolated through a phenol extraction or **(B)** RuBisCO-depletion method were compared across the six time points by 2-D DIGE using 24 cm pH 4–7 (left to right) IPG strips and 12.5% sodium dodecyl sulfate-polyacrylamide gel electrophoresis (SDS-PAGE) gels. Protein spots of interest are numbered. Panel **(A)** is an overlap image of gel no. 1 (Table S1) with the LL25 sample labeled with Cy3 and the LL37 sample labeled with Cy5. Panel **(B)** is an overlap image of gel no. 4 (Table S1) with the LL29 sample labeled with Cy3 and the LL33 sample labeled with Cy5.

In both approaches four biological replicates were obtained for each time point (12 gels for each extraction method; Table S1). We first analyzed protein samples prepared in parallel from the same tissue samples on 1-D SDS-PAGE gels (Figure S1) and then separated the extracts on high resolution 24 cm 2D gels using the CyDye system (Table S1). The phenol–methanol and RuBisCO-depletion methods consistently yielded highly resolution gels with typically ~1350 protein spots resolved in each gel (Figure 1, Figure S2). Gel image analysis and protein spots were quantified using Progenesis same spot software and spots that reproducibly changed in abundance between two samples within the time series with *p* < 0.05 (*n* = 4) were selected for further examination (77 for the phenol-extracted samples and 59 for the RuBisCO-depleted samples; summaries of peptides matching each protein are in Table S2). A substantial set of changes are apparent by the colored overlay of the fluorescence images. Spots of interest were picked manually from post-electrophoretically silver stained preparative gels and in-gel trypsin-digested. Protein identities were analyzed by LC-MS/MS (Figures 1A,B indicated by arrows). MASCOT software (ver. 2.3.02) was used to simultaneously identify proteins.

From the first set of ANOVA-selected differentially-expressed protein spots, visual inspection narrowed further analysis to 71 spots from the phenol-extracted samples, and 40 spots from the RuBisCO-depleted samples which showed circadian-like waveforms (Tables S3, S4). Some peptides were identified in multiple spots, which was likely caused by post-translational modifications that shift the mobility in 2D gels. Typically many proteins of different families were identified within a single gel spot. Spot identity was then assigned to the protein having the highest percentage of total MS spectra coming from that spot (top hit; Tables S5, S6; see Section Methods and Conclusion).

**Figure 2:**
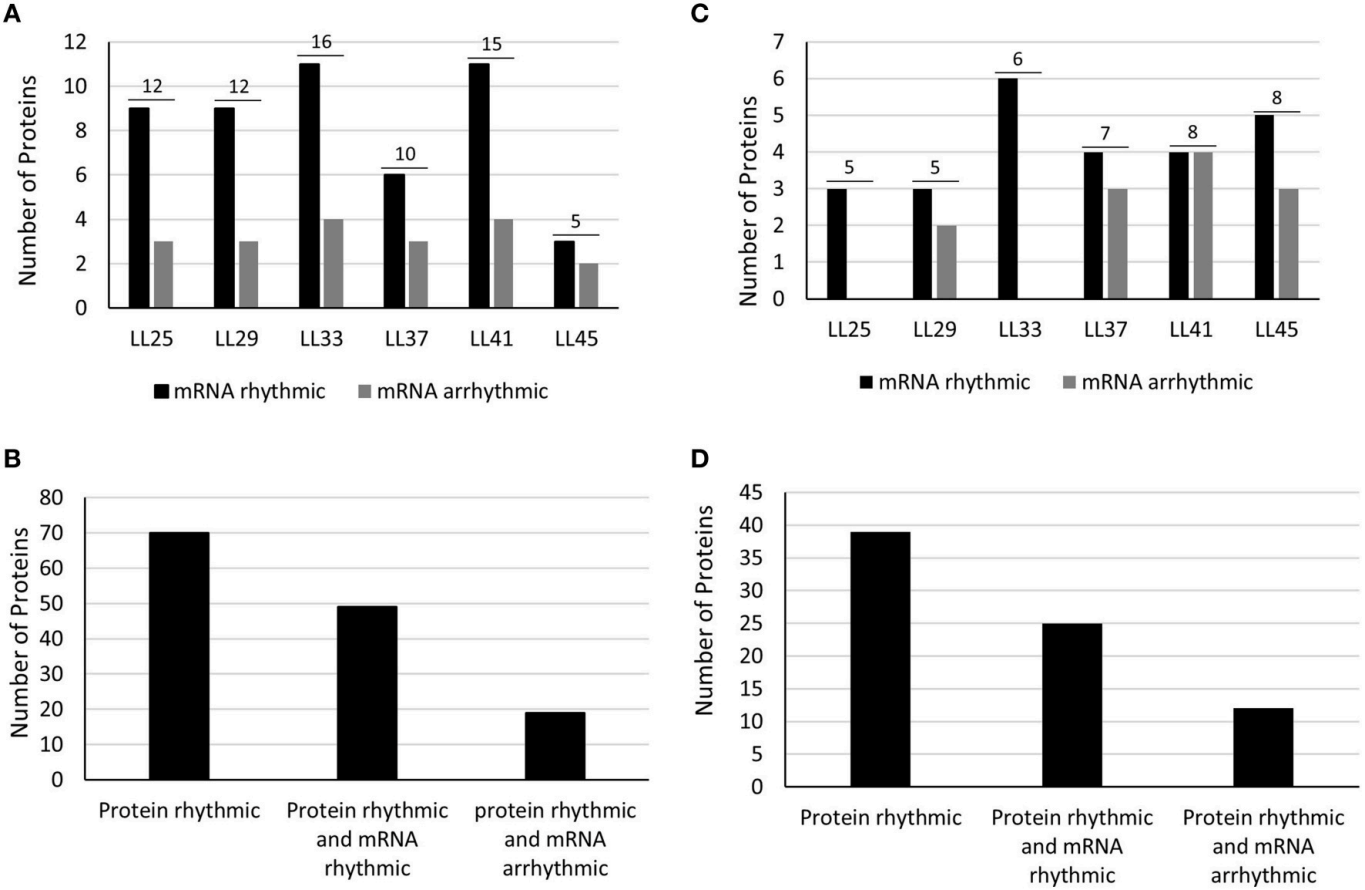
**Protein distribution by circadian phase and mRNA rhythmicity**. Number of rhythmic proteins (Phenol extracted) are shown as the number above the two bars for each phase of peak occurrence **(A)** and relative to whether the respective mRNA is also rhythmic **(B)**. Peak phasing of rhythmic proteins are further shown according to whether the respective mRNA is rhythmic (dark bar) or whether the respective mRNA is arrhythmic (gray bar). **(C,D)** Results for same categories obtained from RuBisCO-depleted protein extracts. Discrepancies between the total number of proteins and the sum of the two bars for each phase is due to mRNA data missing from DIURNAL.

The co-expression profile of the oscillating peptides was represented by a heat map (Figure S3) using the MEV software (The Institute of Genomic Research, TIGR; Saeed et al., 2003). The phase distributions of the cyclic proteins were similar for both datasets, with a similar number of peak phases across the time series (Figures 2A,C). These results differ from a recent circadian phosphoproteome analysis where peak phosphopeptide abundance occurred just after subjective dawn and subjective dusk (Choudhary et al., 2015). mRNA phasing patterns in Arabidopsis were more evenly distributed across day and night, though there was still a slight bias toward near dusk and near dawn (Harmer et al., 2000). Rhythmic proteins from both extraction methods mostly correlated with rhythmic transcripts (56 and 69%; phenol-extracted and RuBisCO-depleted, respectively; Figures 2B,D). Thus, between ca. 30 and 40% of rhythmic proteins derive from non-rhythmic transcripts, and these were not associated with a particular circadian phase (Figures 2B,D). These results are similar to an Arabidopsis phosphoproteomic study which reported that more than half of the cycling phosphopeptides came from genes with arrhythmic transcripts (Choudhary et al., 2015). Similar findings were reported from mouse liver studies, where 20–50% of the rhythmic proteins did not exhibit corresponding rhythmic transcripts (Reddy et al., 2006; Mauvoisin et al., 2014b; Robles et al., 2014). Taken together, these results emphasize the importance of circadian control over post-transcriptional and post-translational processes that lead to the net result of rhythmic patterns of protein abundance.

### Immunoaffinity removal of RuBisCO

**Figure 3:**
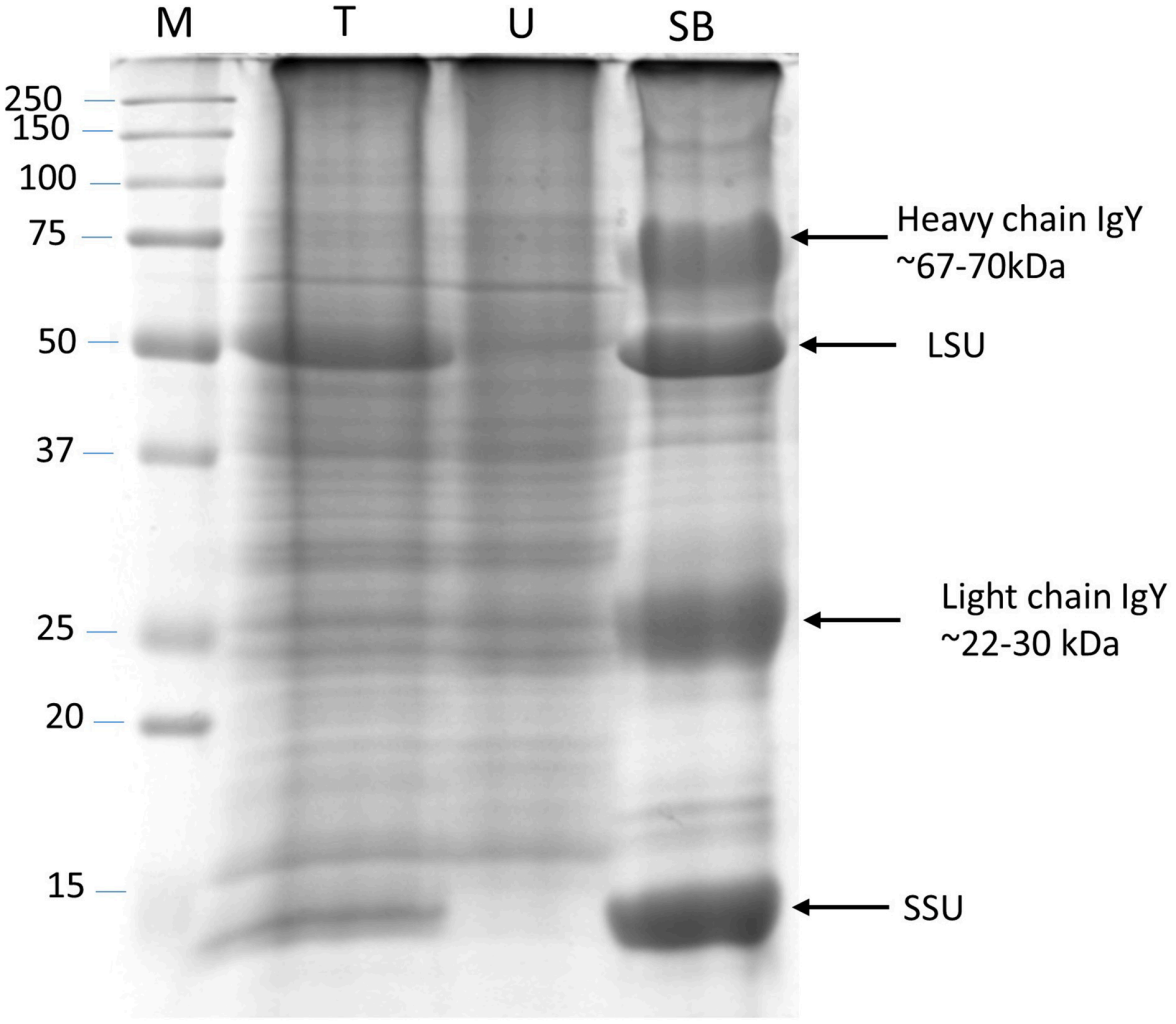
**RuBisCO depletion from total protein extracts**. Silver stained SDS-PAGE (12%) gel showing RuBisCO depletion after immunoaffinity purification using a Seppro IgY-RuBisCO spin column. Large (LSU) and small (SSU) subunit of RuBisCO are marked. M, Molecular weight marker; T, Total protein; U, Unbound protein fraction; SB, Specifically bound proteins.

**Figure 4:**
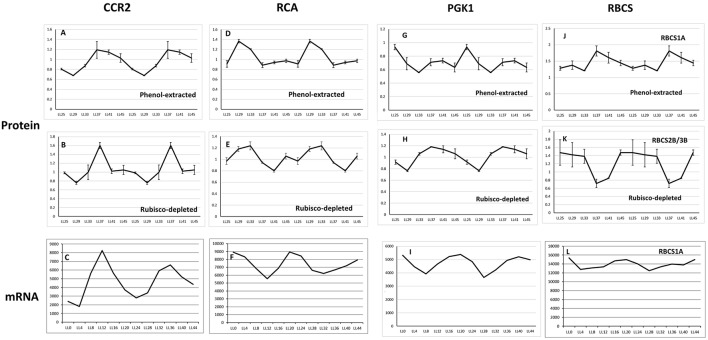
**Expression profile of four oscillating proteins found common to the Phenol-extracted and RuBisCO-depleted data sets**. Protein oscillations for *CCR2* **(A,B)** *RCA* **(D,E)** *PGK1* **(G,H)** and *RBCS* **(J,K)** are double-plotted and comparisons with the transcript profiles of *CCR2* **(C)**, *RCA* **(F)**, *PGK1* **(I)** and *RBCS* **(L)** should begin with the second half of the time series (LL24). Transcript levels from DIURNAL. Error bars show standard deviation from 4 biological trials. The transcript profiles for AT5G38410 and AT5G38420 are not available from the DIURNAL website. See text for details.

RuBisCO-depletion spin columns (Seppro® IgY affinity purification; SIGMA) effectively removed the large and small subunit of RuBisCO (Figure 3; compare boxed areas in Figures S2A,B). One indication of the effectiveness of RuBisCO depletion is the many fewer number of RuBisCO peptides identified in the depleted samples (10) compared to the phenol-extracted samples (45; Table S7). Surprisingly, only four proteins were found common to both data sets as the top hit in the respective spots: cold, circadian rhythm RNA-binding 2 (CCR2/ATGRP7; spot P69 and S59), phosphoglycerate kinase 1 (PGK1; spot P42 and S50), RuBisCO activase (RCA; spot P06, P12 and spot S11), and ribulose-1,5-bisphosphate carboxylase/oxygenase (RuBisCO) small subunit (RBCS; spot P73 and S49; Tables S3, S4; Figure 4). CCR2/ATGRP7 is an RNA-binding protein that is part of a molecular slave oscillator associated with the Arabidopsis circadian system (Schmal et al., 2013). Both extraction methods showed the same late-day peak phase of CCR2 protein (Heintzen et al., 1997), similar to the transcript, but the amplitude of oscillation was more robust using the RuBisCO-depletion extraction method (Figures 4A–C).

**Figure 5:**
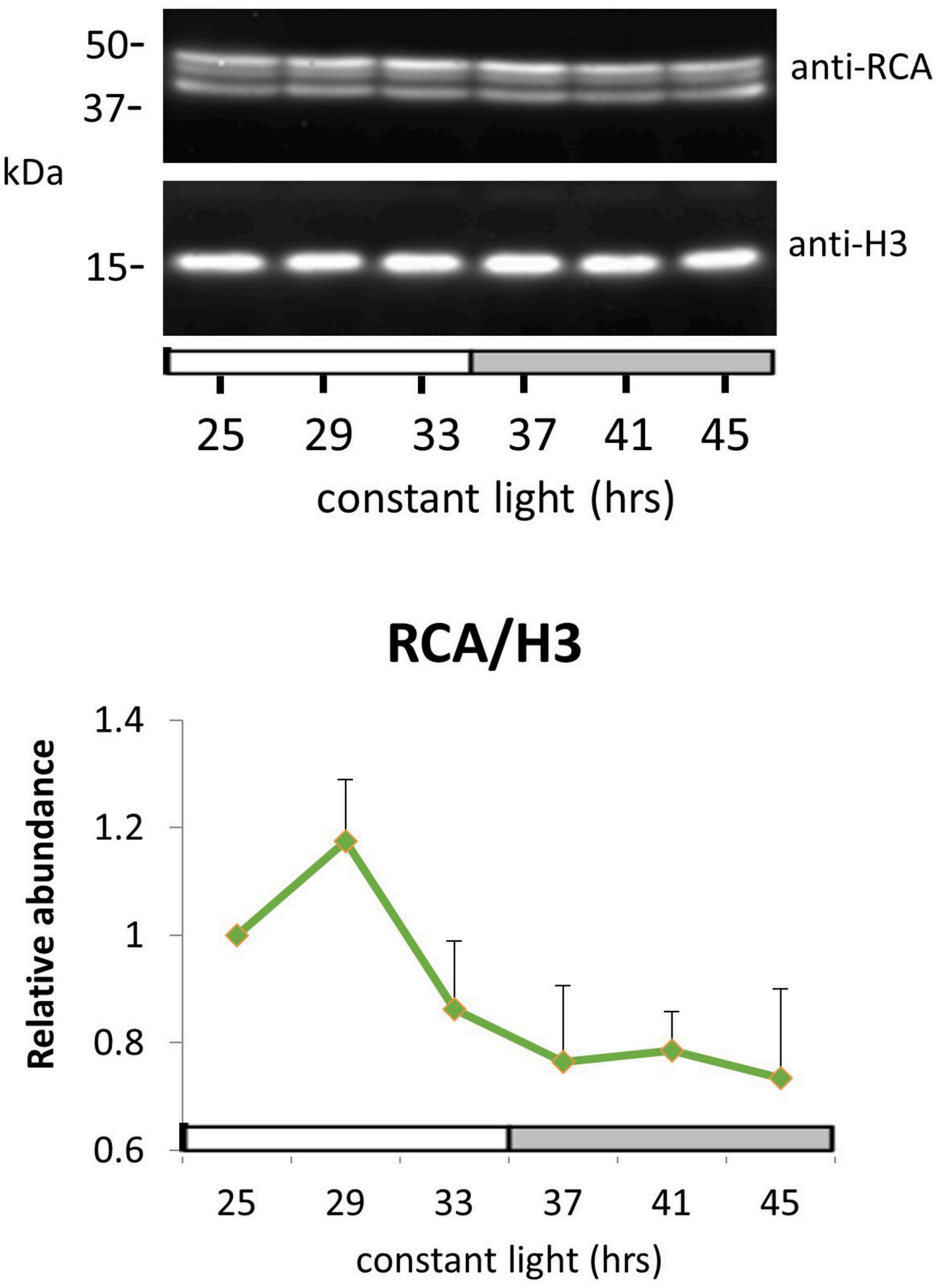
**RuBisCO activase protein oscillation in constant light**. Immunodetection of RuBisCO activase (RCA) protein abundance. Ten-day-old Arabidopsis Col-0 seedlings grown under LD cycles were released to continuous light and harvested at the indicated time. White and gray bars indicate subjective day and night. Protein level was quantitated relative to histone H3 and normalized to the level of LL25. Error bars indicate *s.e.m*. from two biological replicates.

Peak RCA protein abundance was early-day phased for both methods and slightly phase-delayed relative to peak RCA mRNA abundance (Figures 4D–F). We validated these results by immunoblot using whole seedling extracts grown under the same entrainment and free-running conditions. Peak RCA levels occurred at the same phase and with similar amplitude as that obtained from the two 2D-DIGE data sets (Figure 5). Both approaches resolved oscillations with a two-fold or less range in abundance, indicating a high degree of sensitivity and reproducibility using 2D-DIGE/MS.

PGK1 is an essential enzyme that catalyzes the reversible ATP-dependent phosphorylation of 3-phosphoglycerate (3-PGA) in the chloroplast, as well participating in the glycolytic pathway in the cytosol (Morisse et al., 2014). PGK1 protein oscillations from the RuBisCO-depleted samples were phased very similar to the transcript (Figures 4G–I).

Two to three isoforms of the RuBisCO small subunit (RBCS3B/RBCS2B and RBCS1A) showed different phases of peak oscillation (Figures 4J–L). RBCS3B/RBCS2B (spot S49; Table S4) maximally accumulated during subjective day, dropping to lowest levels in the early evening. RBCS1A (spot P73; Table S3) was anti-phasic to 2B/3B, rising to maximal levels at subjective dusk. The significance of these two forms of RuBisCO peaking at different times is unclear. Interestingly, RBCS1A expression is highest in plants grown at low temperatures, with levels dropping in plants grown at higher temperatures. However, RBCS3B/RBCS2B expression rises strongly with increasing growth temperatures (Yoon et al., 2001). Further work on the phasing and oscillation amplitude of these RBCS proteins in plants grown at different temperatures could be very informative.

### GO analysis

**Figure 6:**
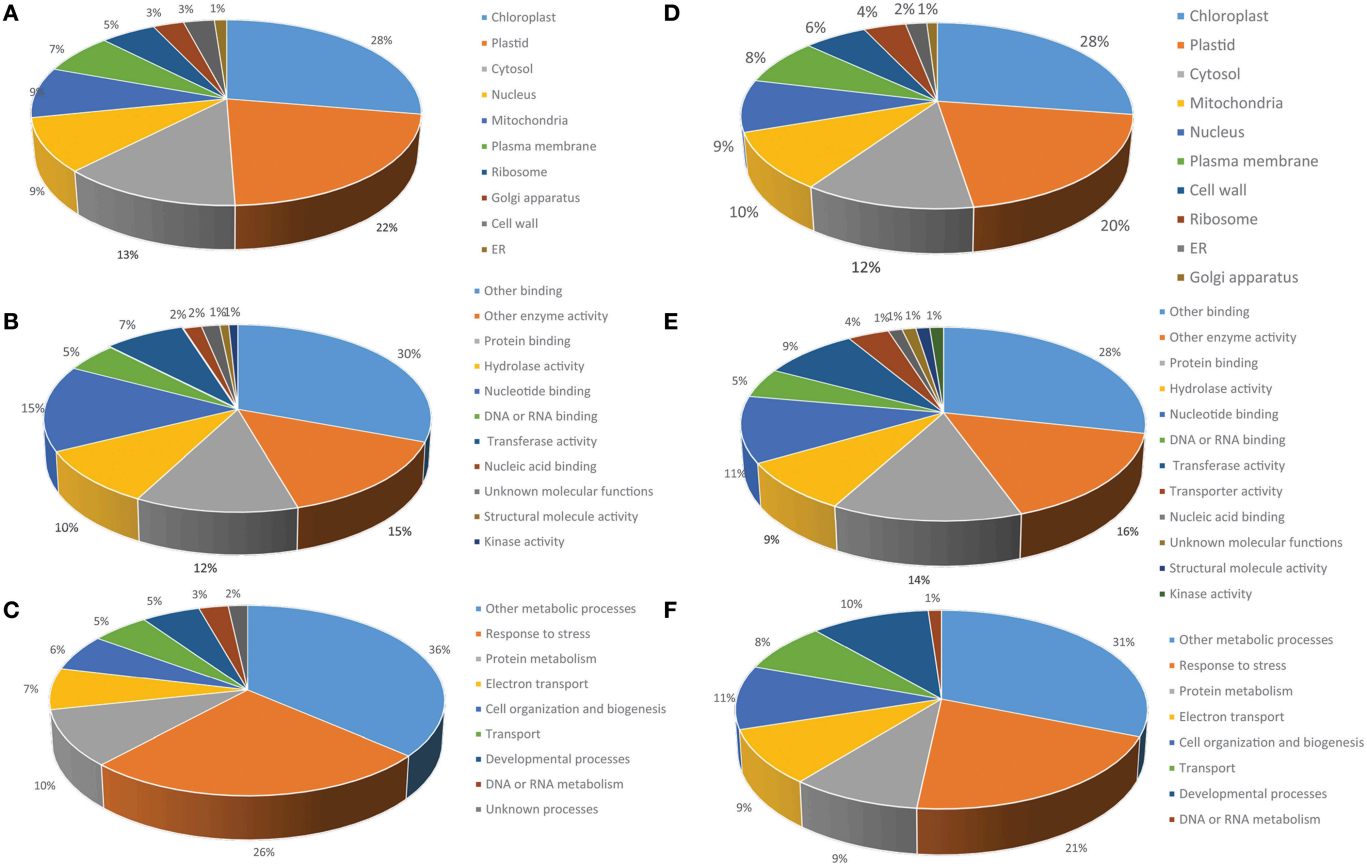
**Functional classification of the phenol-extracted circadian-regulated proteins by GO analysis**. Percentage GO terms were displayed for three GO vocabularies: **(A)** Cellular component, **(B)** Biological process, and **(C)** Molecular function. **(D–F)** Results for same categories obtained from RuBisCO-depleted protein extracts.

For further characterization we performed GO analysis using TAIR Slim GO for cellular component, molecular function and biological process. The cellular component representation is extremely similar for both the protein extraction procedures, but there is slightly less representation of chloroplast and plastid protein in the RuBisCO-depleted extracts (48 vs. 50%), possibly reflecting the removal of RuBisCO (Figure 6A). Within the molecular function category, proteins involved in enzymatic activity and protein and nucleotide binding are the major players in both data sets. At the biological process level proteins related to stress response and protein metabolism are the major contributors (Figure 6). Thus, the two types of extractions displayed a high degree of similarity in the relative contribution of three different GO vocabularies examined.

### String analysis

**Figure 7:**
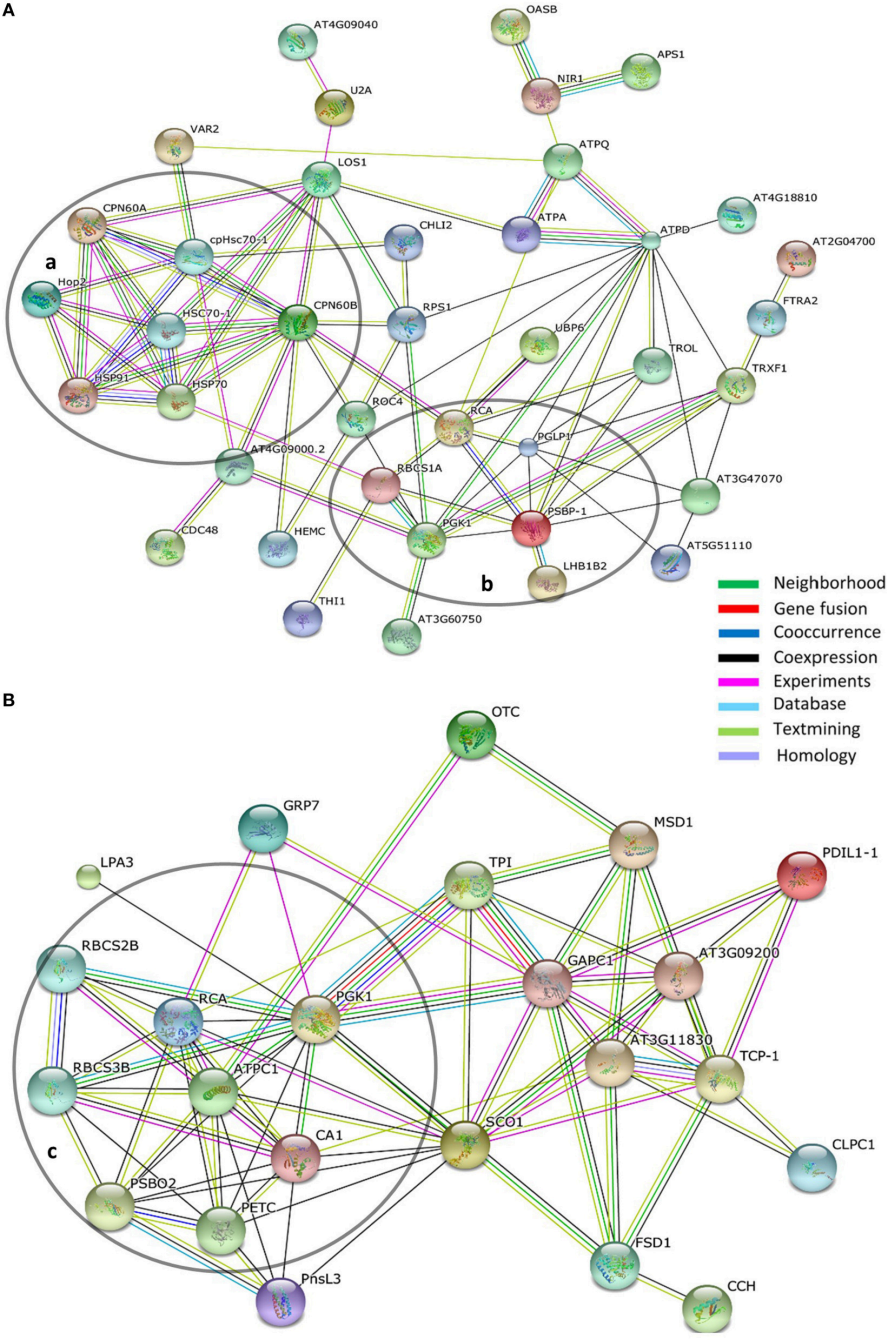
**Interaction networks within circadian protein data sets. (A)** Phenol-extracted and **(B)** RuBisCO-extracted data sets. Network mapping performed using the STRING protein interaction algorithm based on the STRING database using high confidence parameters. Proteins not connected with network were removed for better visualization. Line colors indicate the types of evidence for each association. Circled groupings indicate (a) chaperone-related and (b,c) photosynthesis-related networks.

The STRING database allows accumulated protein-protein interaction data to be assembled and viewed as interaction networks (Szklarczyk et al., 2015). Using STRING we identified and focus on groupings of rhythmic proteins associated with photosynthesis (the phenol-extracted and RuBisCO-depleted data sets) and chaperones (phenol-extracted data set; Figure 7).

### Photosynthesis

Taken together the two proteome data sets identified 10 proteins or protein families associated with photosynthesis that oscillate with a circadian period (Figures 7A,B). As noted above, members of the RBCS protein family, RCA and PGK1 were common to both approaches. Two proteins involved in the regulation of photosystem II appear in more than one spot and with different peak phases (PSBO2; S22, S23, S25, and PSBP-1; P16, P22; Tables S3, S4). Both undergo post-translational modifications (PhosPhAt 4.0; Zulawski et al., 2013), which may account for the same polypeptides migrating into different gel spot positions. The different peak phasings among the spots are intriguing and suggest a change in the extent of the modification within each protein (likely phosphorylation; Zulawski et al., 2013) over circadian time (Tables S3, S4).

Two proteins with nearly identical profiles, PETC/PGR1 (spot S42; Table S4) and ATPC1 (spot S13; Table S4), show maximum accumulation at LL 45, 3 h before subjective dawn. PETC/PGR1 is a component of the cytochrome b6-f complex and confers resistance to photo-oxidative damage (Maiwald et al., 2003). ATP synthase gamma chain 1 (ATPC) is an essential element of the light-dependent regulation of chloroplast ATP synthase activity (Wu et al., 2007). It is intriguing that under constant light the maximum accumulation of both proteins occurs well before the anticipated time of dawn. It is possible that under normal dark/light conditions the onset of light acutely induces an additional boost in accumulation of both proteins, which would be additive to the high baseline established by circadian system in the dark. This would enhance, during the daylight hours, the levels of both proteins which are so necessary to photosynthesis.

### Chaperones

Both proteome data sets identified circadian oscillations in chaperone levels. The plastid-localized chaperonin 60 alpha2 and beta3 (Cpn60α2 and Cpn60β3) were identified in the phenol-extracted data set (Table S3). Cpn60 is found in the stroma and is instrumental in the maturation of RuBisCO large subunit (RbcL), RuBisCO activase (RCA), and other enzymes associated with photosynthesis (Trösch et al., 2015). The two most robust instances of Cpn60α2 and Cpn60β3 cycling (P03 and P10; Table S3) are maximally phased at mid to late in the day phase. This is very similar to the phasing of the stroma-localized cpHSC70-1 (P02; Table S3). cpHSC70s are essential to chloroplast development and are key to the import of plastid stromal proteins (Latijnhouwers et al., 2010; Su and Li, 2010; Flores-Pérez and Jarvis, 2013).

ClpC/Hsp93-V was identified in the RuBisCO-depleted data set (S01, S02; Table S4). This chaperone is also involved in plastid protein import, particularly with the TOC/TIC (translocon at the outer/inner chloroplast envelope membrane) complex (Flores-Pérez and Jarvis, 2013). Both isolates were phased with highest presence during the early and mid-day. Taken together, the similar phasing of maximal protein expression of these three plastid-associated chaperones (Cpn60, cpHSC70, and ClpC/Hsp93-V) indicate the importance for strong circadian coordination of plastid protein import and maturation.

Additionally, the cytosolic co-chaperone Hop2 (HSP70 and HSP90 organizing protein) was rhythmically expressed with a morning peak phased slightly earlier than the plastid-associated chaperones (P29, P32; Table S3). Hop can bind to HSP90 and HSP70 simultaneously and acts to facilitate the transfer of nascent client proteins through the intermediate steps that lead to the fully mature client (Zhang et al., 2003; Baindur-Hudson et al., 2015). Interestingly, the Hop co-chaperone has been found in a large complex in association with HSP90 and various chloroplast preproteins (Fellerer et al., 2011). This finding, together with the oscillations in Hop2 levels (Table S3) suggests that maturation of chloroplast-bound proteins, prior to plastid entry, is also under circadian clock control.

HSP70 is well-established as an early-interacting partner in HSP70/HSP90-dependent protein maturation in a wide range of processes in plants and animals (Sung and Guy, 2003; Jung et al., 2013; Radons, 2016). HSP70 was recovered from four different spots in the phenol-extracted data set (P01, P07, P30, and P31; Table S3) which correspond to two cytosolic forms, HSP70-1/HSC70-1 and HSP70-4 (Lin et al., 2001). Both forms showed early to mid-day peaks in protein oscillations that matches well with peak Hop2 expression (Table S3). The phasing coincidence of these key players in protein maturation adds further support to the notion of circadian regulation in the formation/activity of a HSP70–Hop2–HSP90 complex that processes chloroplast preproteins.

We previously reported robust oscillations in the phosphorylation state of HSP70-1/HSC70-1 (Choudhary et al., 2015). Comparison of HSP70-1/HSC70-1 protein oscillation with its phospho-oscillation indicates a slightly earlier peak in protein levels (LL 33; Table S3) compared to the phosphorylation peak (LL 37). This may indicate that much of the oscillation in HSP70-1/HSC70-1 phosphorylation follows from circadian-driven changes in its protein levels.

There are a number of ways the nuclear-based circadian system exerts control over the chloroplast (Atkins and Dodd, 2014). The clock-regulated nuclear-encoded sigma factor SIG5 can confer oscillations on certain chloroplast-encoded transcripts, demonstrating one level of nuclear control over chloroplast function (Noordally et al., 2013). Direct circadian regulation of tetrapyrolle biosynthesis gene expression and chlorophyll-binding protein gene expression also contribute to chloroplast assembly and function (Millar and Kay, 1991; Matsumoto et al., 2004). Our above results linking clock control of chaperone-mediated protein maturation now reveal an additional level of circadian control to chloroplast assembly and photosynthesis activity.

### Abscisic acid (ABA) signaling

Of special note is the distinct circadian oscillation in the abundance of the ABA receptor (PYR1/RCAC11, S37; Table S4; Figure S4). The peak levels occur just after subjective dusk (LL 41) and does not track mRNA levels. DIURNAL (Mockler et al., 2007) reports no apparent mRNA oscillation for the first 24 h in constant light (Table S4), but a there is a weak oscillation with peak transcript level near LL28-29 (Table S4; Figure S4), anti-phase with the protein peak. This finding is striking in the context of other known levels of clock control over the ABA system.

The ABA signaling pathway begins with three type of components, the ABA receptor (PYR/RCAC proteins), protein phosphatase 2C (PP2C), and sucrose nonfermenting-1 (SNF1)-related protein kinase 2 (SnRK2; Yoshida et al., 2015). The downstream signaling initiated by PYR/RCAC binding of ABA strongly relies on the phosphorylation of numerous substrates by SnRK2s at many stages of plant development (Yoshida et al., 2015). A previous phosphoproteomic study identified subjective dusk/early evening (LL37-45) peak phosphorylation of two SnRK2 proteins (SNRK 2.2 and SNRK 2.3), the ABA-regulated transcription factor AREB1/ABF2 and ABA-responsive gene COR78/RD29a (Choudhary et al., 2015). An ABA/PYR1/PP2C complex activates SnRK2; hence the similar circadian phasing of the receptor and kinase components will accentuate systemic responsiveness to ABA levels in a late-day phase-dependent way. As well, ABA levels tend to peak in the late afternoon/early evening under light/dark cycles, further heightening ABA signaling potential at this time (Nováková et al., 2005; Lee et al., 2006; Fukushima et al., 2009). Circadian regulation of the transcript abundance of numerous ABA signaling components (e.g., *SnRK2.6, ABI1, ABF3, RCAR1*, and others Seung et al., 2012) that are similarly dusk/early evening phased highlights the importance of ABA responsiveness late in the day.

Further, there is a complex interaction between the core circadian system and ABA-responsive factors. TOC1 binds to the promoter of the ABA-related gene *ABAR/CHLH/GUN5*, repressing its expression and conferring a circadian pattern to *ABAR* transcript accumulation. Reciprocally, *TOC1* expression increases in response to ABA, which requires the presence of *ABAR* (Legnaioli et al., 2009; Pokhilko et al., 2013). The TOC1-related protein, PRR7, is similarly required for correct phasing of ABA-mediated gene expression (Liu et al., 2013). Additionally, the clock-regulated ABA-inducible R2R3-type MYB transcription factor, MYB96, binds to the TOC1 promoter affecting clock-dependent gating of ABA responses (Lee et al., 2016). The addition of a circadian oscillation in ABA-receptor (PYR1) levels now adds further complexity to the regulation of these downstream interrelationships.

## Conclusions

Through 2D-DIGE, coupled with mass spectrometry, we surveyed the Arabidopsis seedling proteome for circadian-regulated proteins using two different protein extraction techniques. We supplemented a standard phenol-extraction approach with an immuno-depletion technique in an effort to identify low abundance proteins that might be masked by the overwhelming presence of RuBisCO. Among the proteins which cycled in abundance, 287 proteins were found overlapping between the two methods (Table S7) and only four emerged as similar top hits within their respective spots (Figure 4). These results indicate that the apparent discrepancies between the datasets do not come from two very different sets of results, but arise more from differences in the relative abundances among the many proteins recovered that are common to both extraction methods. From this perspective, the two extraction approaches were complementary and together enhanced our discovery of novel circadian-regulated proteins.

Two-dimensional electrophoresis (2-DE) does not have the resolution to separate all proteins into one single spot for each. Generally each spot contains more than one protein and the same protein may be detected from different spots due to post-translational modifications or proteolytic cleavage. The number of proteins detectable from any spot depends on the method used for identification, e.g., MALDI-TOF can detect fewer peptides than LC-MS/MS. In the present study we used an LTQ-Orbitrap XL, the most advanced MS technique, which enhanced detectability. Hence, more than one protein per spot is often detected (Thiede et al., 2013).

For each spot we selected the top hit or protein as the one having the most spectra, assuming that the top hit protein is the major contributor to spot pattern determination. It is practically not feasible to select a spot from each of the six analytical gels and analyze that same spot for each of the six time points. Hence, pooled samples were used for the preparative gels (see Section Methods). It is possible that the same non-cycling protein present at a given spot over the time series could obscure the oscillations of a less abundant protein, which will reduce recovery of low abundance, oscillating proteins. But this would likely show up as the top hit, but not be processed because we only analyzed spots showing oscillations. This same occurrence could also simply dampen the amplitude of a more dominant oscillating protein. Since all the time points were pooled for MS only proteins aberrantly very high at one or two time points could emerge as the top hit but not be the same protein responsible for the oscillating pattern. As well, multiple proteins with similar abundances within a spot could obscure or alter the waveform of an oscillating protein; in these cases interpretations must proceed with caution. In instances where the scores are very similar or identical we consider both the MW and PI for spot identity or if both parameters are similar we show both proteins as candidates (e.g., P77, S18; Tables S5, S6).

Often oscillations in protein levels reflect transcript rhythms, but with a phase delay of 2–3 h or more (Fujiwara et al., 2008; Nusinow et al., 2011; Rawat et al., 2011; Robles et al., 2014). Our results here, and previously (Choudhary et al., 2015), show that more than 35% of the rhythmic proteins lack a corresponding rhythmic transcript, in line with studies in other circadian systems (Reddy et al., 2006; Deery et al., 2009; Mauvoisin et al., 2014b; Robles et al., 2014). Clearly, a significant number of circadian-regulated proteins are being missed by a reliance on transcript oscillation as an indicator of polypeptide rhythms. Recent findings demonstrate that these rhythms may arise through circadian control of protein synthesis/translation (Künne et al., 1998; Jang et al., 2015; Janich et al., 2015; Missra et al., 2015; Feeney et al., 2016), protein turnover (Kim et al., 2003, 2007, 2011; van et al., 2011; Yoo et al., 2013; Stojkovic et al., 2014; DeBruyne et al., 2015), or a combination of the two. The circadian regulation of proteostasis appears underappreciated as a mechanism to control polypeptide levels. Our results will help initiate further investigations into the post-translational regulation of pathways and processes regulated by the Arabidopsis circadian system.

## Author contributions

MC, HS, HN, and DS designed research; MC, YN, HS, and HN performed research; HN contributed new reagents or analytic tools; MC, HS, HN, and DS analyzed data; MC and DS wrote the paper.

## Funding

This work was supported by National Institutes of Health Grant R01GM093285 (to DS) and the World Class University Program of South Korea (No. R31-2008-000-10105-0), NRF, MEST (to DS), and by JSPS KAKENHI Grant Numbers 26650106 and 15H01247 to HN.

### Conflict of interest statement

The authors declare that the research was conducted in the absence of any commercial or financial relationships that could be construed as a potential conflict of interest.
