# Supplementary material for: Circadian Profiling of the Arabidopsis Proteome Using 2D-DIGE
Source: Front Plant Sci. 2016 Jul 12;7:1007. doi: 10.3389/fpls.2016.01007 (PMC4940426; doi:10.3389/fpls.2016.01007)
Supplement: Supplementary file 1 [file Table1.PDF]

Gel No.	Cy2	Cy3	Cy5
1	IS	LL25(1)	LL37(2)
2	IS	LL41(3)	LL29(1)
3	IS	LL33(2)	LL41(4)
4	IS	LL29(2)	LL33(3)
5	IS	LL41(4)	LL37(3)
6	IS	LL25(2)	LL41(2)
7	IS	LL37(1)	LL45(2)
8	IS	LL29(4)	LL41(1)
9	IS	LL41(3)	LL33(1)
10	IS	LL45(1)	LL29(4)
11	IS	LL29(3)	LL25(4)
12	IS	LL37(4)	LL25 (3)

Table S1. **Design of 2-D DIGE experiments for biological and technical replication.** Each sample was covalently labelled with a different fluorophore, Cy2 (a mixture of equal amounts of protein extracts from all the time points : IS internal standard), Cy3, and Cy5. Parentheses indicate how the time point harvests from the four biological trials were paired among the 12 gels.
