# Supplementary material for: Circadian Profiling of the Arabidopsis Proteome Using 2D-DIGE
Source: Front Plant Sci. 2016 Jul 12;7:1007. doi: 10.3389/fpls.2016.01007 (PMC4940426; doi:10.3389/fpls.2016.01007)
Supplement: Supplementary file 3 [file Table3.PDF]

Table S3: Expression profiles of rhythmic proteins and corresponding mRNA under constant light (phenol extraction method)

Sample No.[1]	Protein ID[2]	Gene locus[3]	Thr MW/ Exp MW[4]	% of spectra	No. of unique peptide	No. of unique spectra	No. of total spectra	% Sequence Coverage	Protein expression profile[5]	Transcript profile[6] (Diurnal)
P01	HSC70-1, HSP70-1, AT-HSC70-1, HSC70 | heat shock cognate protein 70-1	AT5G02500.1	72.00/71.35	11.20	23	40	179	17.20	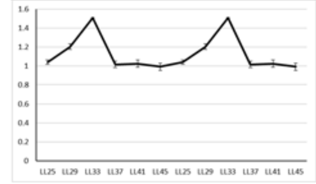	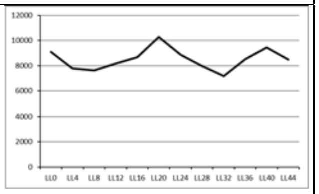
P02	cpHsc70-1 | chloroplast heat shock protein 70-1	AT4G24280.1	76.00/76.50	16.30	56	82	272	63.90	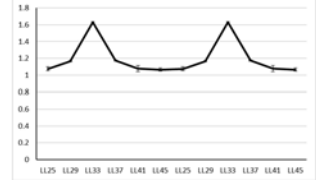	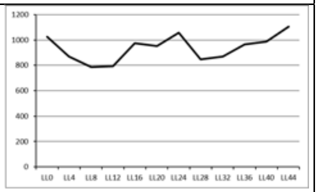
P03	CPN60A, CH-CPN60A, SLP | chaperonin-60 alpha	AT2G28000.1	63.00/62.07	18.50	49	72	249	80.70	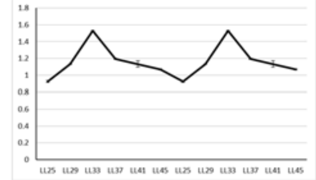	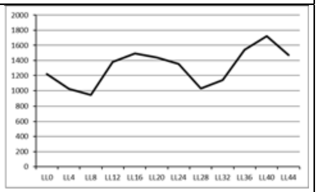
P04	TROL | thylakoid rhodanese-like	AT4G01050.1	59.00/49.38	6.08	9	12	24	15.20	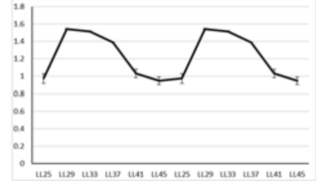	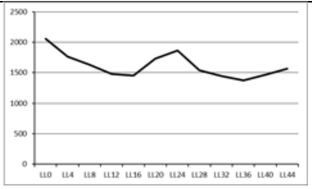
P05	Transketolase	AT3G60750.1	76.00/79.97	15.80	35	51	201	62.20	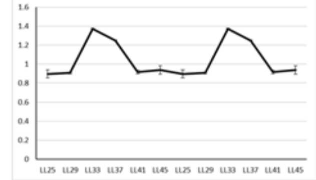	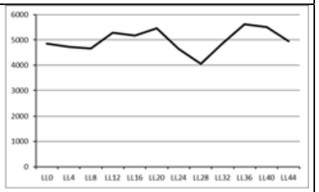

P06	RCA | rubisco activase	AT2G39730.1	76.00/51.98	10.20	17	24	153	47.50	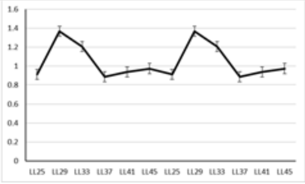	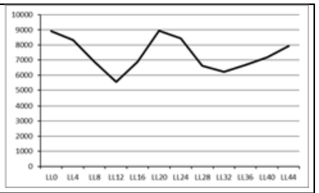
P07	HSP70, ATHSP70-4 | heat shock protein 70	AT3G12580.1	72.00/71.10	12.90	42	69	184	67.70	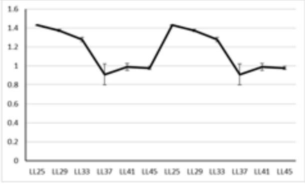	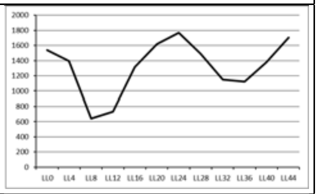
P08	VAR2, FTSH2 | FtsH extracellular protease family	AT2G30950.1	69.00/74.15	8.00	24	32	60	35.40	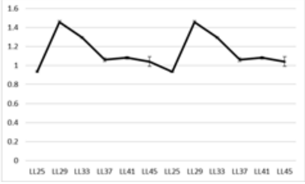	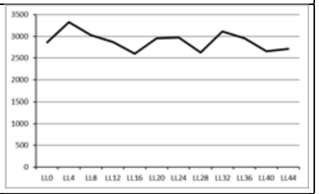
P09	ATPA | ATP synthase subunit alpha	ATCG00120.1	60.00/55.32	8.61	20	31	74	38.50	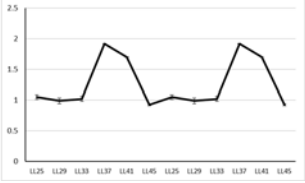	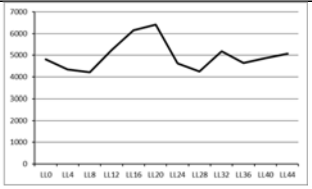
P10	CPN60B, LEN1 | chaperonin 60 beta	AT1G55490.1	62.00/63.80	22.40	48	71	367	74.50	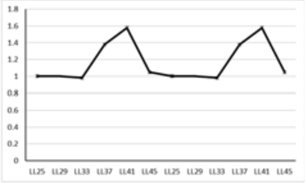	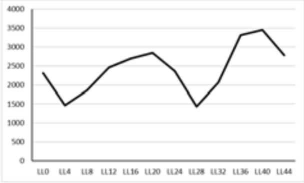
P11	ATPA | ATP synthase subunit alpha	ATCG00120.1	59.00/55.32	8.62	32	46	135	56.40	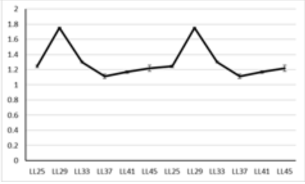	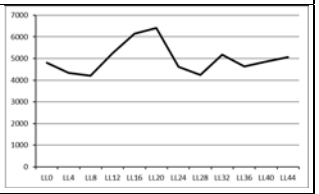

P12	RCA | rubisco activase	AT2G39730.1	47.00/51.98	35.40	23	37	313	62.00	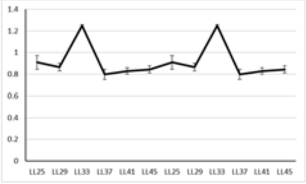	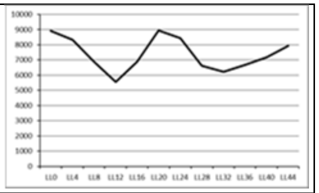
P13	VLN2, ATVLN2 | villin 2	AT2G41740.1	95.00/107.84	12.80	29	37	71	39.30	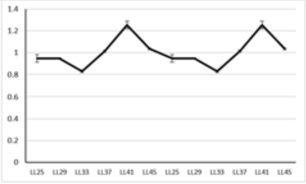	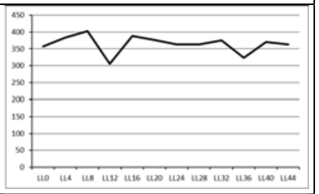
P14	VAR2, FTSH2 | FtsH extracellular protease family	AT2G30950.1	34.00/74.15	6.04	15	22	43	25.80	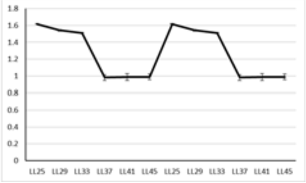	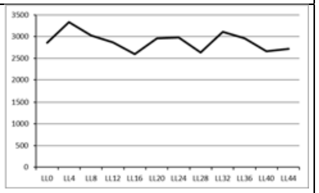
P15	LHB1B2, LHCB1.5 | photosystem II light harvesting complex gene B1B2	AT2G34420.1	26.00/28.05	3.17	8	13	43	44.90	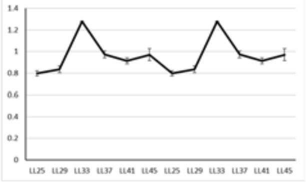	No Data
P16	PSBP-1, OEE2, PSII-P, OE23 | photosystem II subunit P-1	AT1G06680.1	25.00/28.07	5.99	8	12	63	38.00	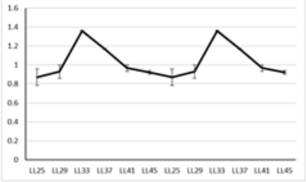	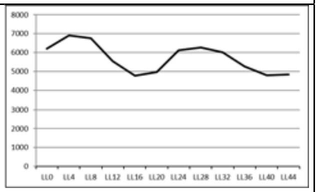
P18	HSP91 | heat shock protein 91	AT1G79930.1	89.00/91.75	23.10	34	47	121	53.40	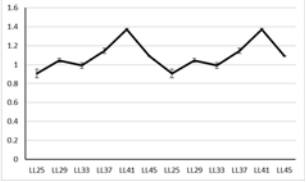	No data

P19	CDC48, ATCDC48, CDC48A | cell division cycle 48	AT3G09840.1	88.00/89.39	13.70	33	39	107	48.70	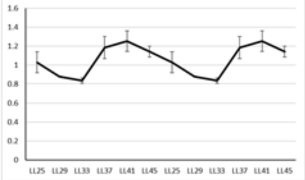	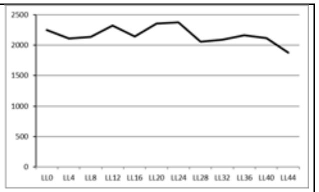
P20	DPE2 | disproportionating enzyme 2	AT2G40840.1	87.00/109.78	11.00	51	69	140	50.10	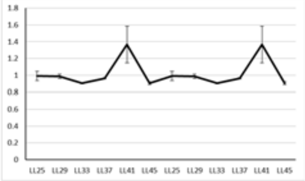	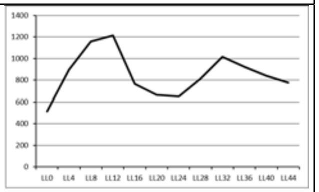
P21	LOX2, ATLOX2 | lipoxygenase 2	AT3G45140.1	86.00/102.04	11.10	44	58	141	47.40	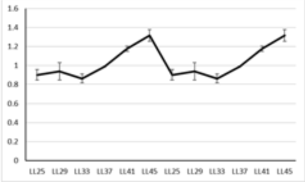	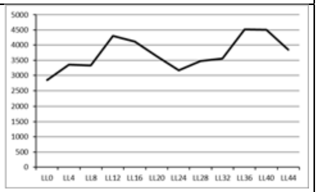
P22	PSBP-1, OEE2, PSII-P, OE23 | photosystem II subunit P-1	AT1G06680.1	84.00/28.09	5.49	10	14	62	42.20	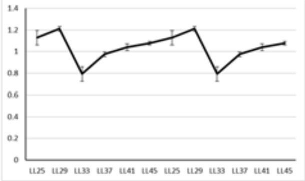	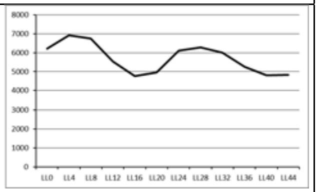
P23	NAI2 | DNA topoisomerase-related	AT3G15950.1	84.00/85.01	25.90	55	74	303	66.60	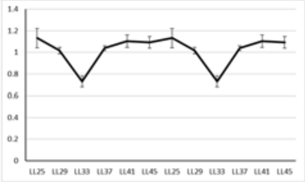	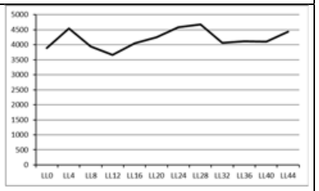
P25	LOS1 | Ribosomal protein S5/Elongation factor G/III/V family protein	AT1G56070.1	83.00/93.89	6.21	28	33	56	43.90	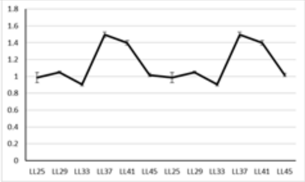	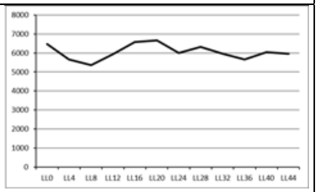

P27	LOX2, ATLOX2 | lipoxygenase 2	AT3G45140.1	76.00/102.04	4.40	28	35	66	39.20	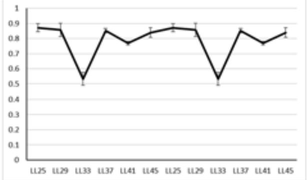	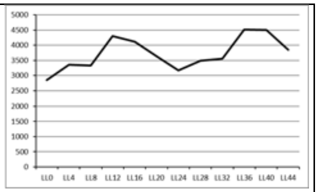
P28	Zincin-like metalloproteases family protein	AT5G10540.1	76.00/79.04	7.27	39	52	108	60.80	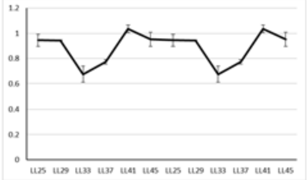	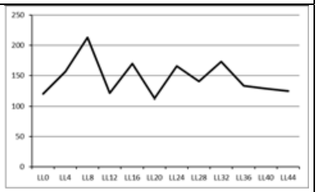
P29	Hop2 | stress-inducible protein, putative	AT1G62740.1	73.00/64.52	6.94	41	59	119	71.10	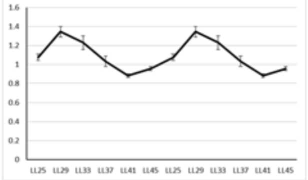	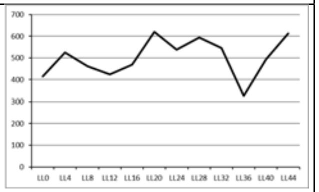
P30	HSP70, ATHSP70-4 | heat shock protein 70	AT3G12580.1	73.00/71.10	7.92	22	32	57	40.60	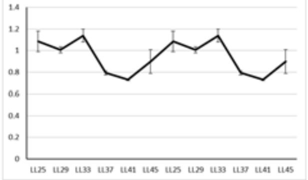	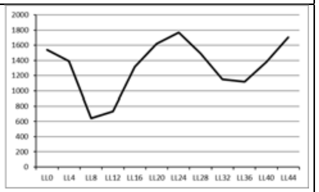
P31	HSP70, ATHSP70-4 | heat shock protein 70	AT3G12580.1	73.00/71.10	14.40	46	72	212	68.60	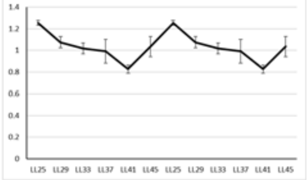	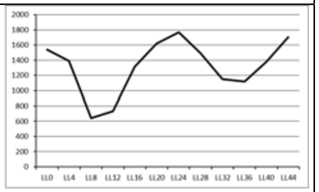
P32	Hop2 | stress-inducible protein, putative	AT1G62740.1	72.00/64.52	6.62	32	43	91	59.70	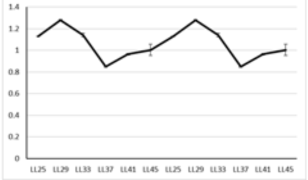	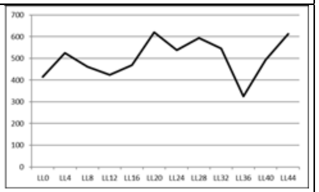

P33	NIR1, NIR, ATHNIR | nitrite reductase 1	AT2G15620.1	68.00/65.50	10.50	40	59	149	64.20	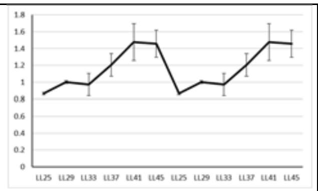	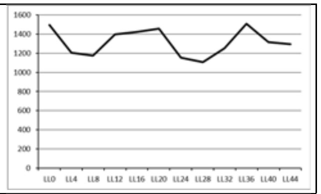
P34	UBP6, ATUBP6 | ubiquitin-specific protease 6	AT1G51710.1	64.00/53.69	6.92	19	28	84	51.90	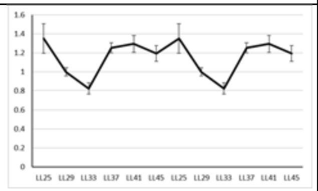	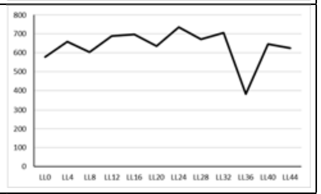
P35	CPN60A, CH-CPN60A, SLP | chaperonin-60alpha	AT2G28000.1	63.00/62.07	8.63	30	41	91	66.40	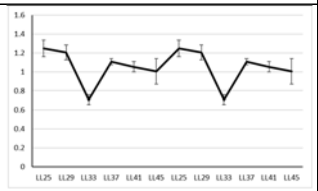	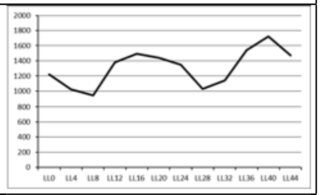
P36	NAD(P)-binding Rossmann-fold superfamily protein	AT4G18810.1	58.00/65.46	5.57	14	18	35	30.40	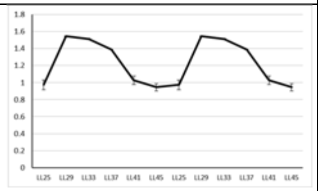	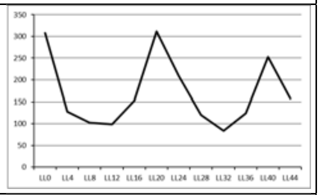
P38	APS1 | ATP sulfurylase 1	AT3G22890.1	47.00/51.46	5.33	27	35	77	57.50	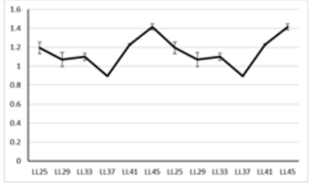	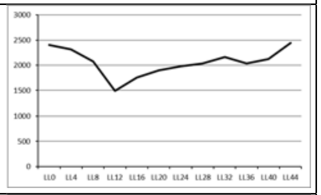
P40	APS1 | ATP sulfurylase 1	AT3G22890.1	46.00/51.46	5.17	24	34	93	54.20	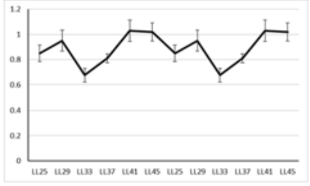	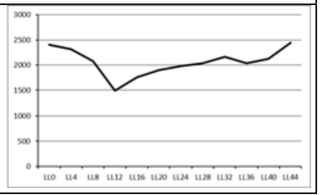

P41	RPS1, ARRPS1 | ribosomal protein S1	AT5G30510.1	46.00/45.11	3.73	21	26	52	47.40	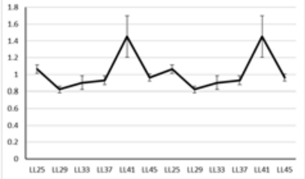	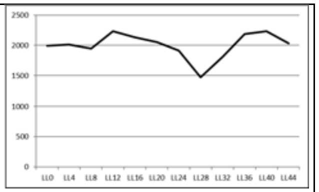
P42	PGK1 | phosphoglycerate kinase 1	AT3G12780.1	45.00/50.11	5.82	25	37	75	73.80	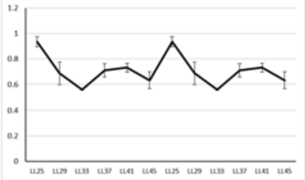	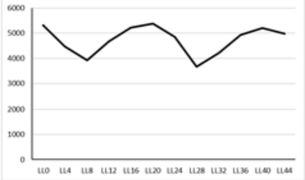
P43	cICDH | cytosolic NADP+-dependent isocitrate dehydrogenase	AT1G65930.1	45.00/45.74	6.95	38	54	133	82.40	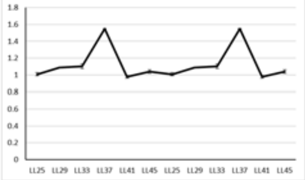	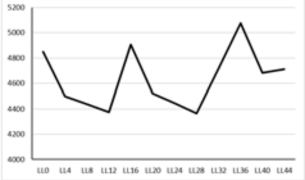
P44	ATMDAR2 : Pyridine nucleotide-disulphide oxidoreductase family protein	AT5G03630.1	44.00/47.48	5.03	26	39	89	74.70	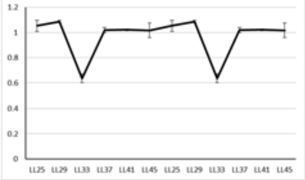	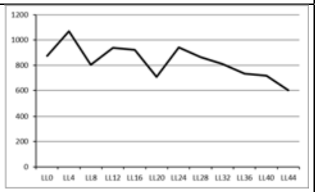
P45	CHLI2, CHL I2, CHLI-2 | magnesium chelatase i2	AT5G45930.1	42.00/46.09	4.89	22	26	67	58.90	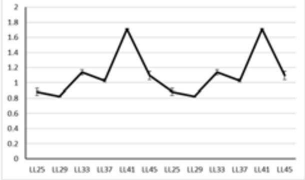	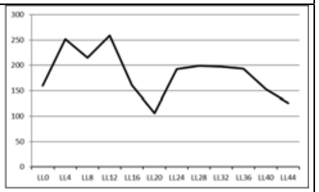
P46	ERD14 : Dehydrin family protein	AT1G76180.1	38.00/20.78	3.11	18	25	44	64.30	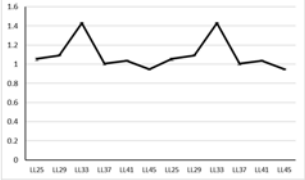	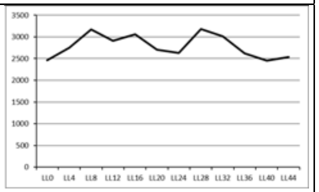

P47	HEMC : hydroxymethylbilane synthase	AT5G08280.1	36.00/41.04	5.20	23	34	78	59.90	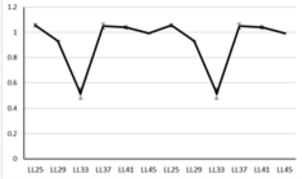	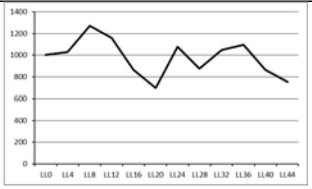
P48	OASB, ACS1, CPACS1, ATCS-B : O-acetylserine (thiol) lyase B	AT2G43750.1	36.00/41.65	4.04	22	26	54	74.00	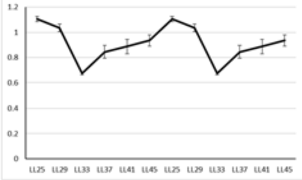	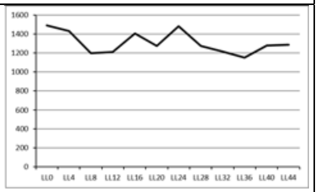
P49	unknown protein; LOCATED IN: endoplasmic reticulum, plasma membrane;	AT1G65270.1	41.00/41.65	2.58	14	21	43	47.30	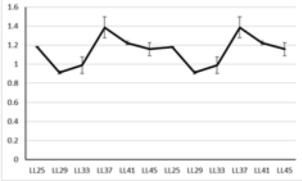	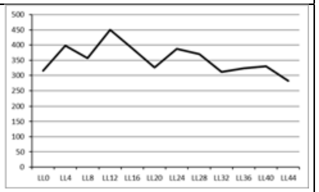
P50	RNA-binding (RRM/RBD/RNP motifs) family protein	AT4G09040.1	34.00/41.65	4.21	14	22	66	47.00	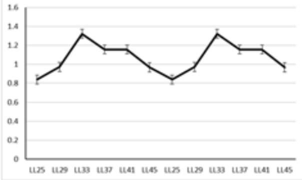	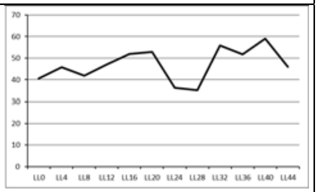
P51	ATPGLP1, PGLP1 : 2-phosphoglycolate phosphatase 1	AT5G36700.1	33.00/39.76	4.52	18	25	58	47.50	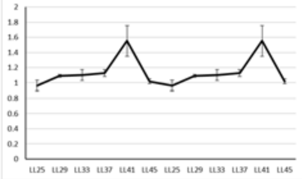	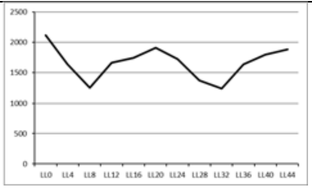
P52	THI1, TZ, THI4 : thiazole biosynthetic enzyme, chloroplast (ARA6) (THI1) (THI4)	AT5G54770.1	32.00/36.66	10.70	14	21	144	62.50	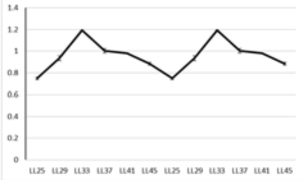	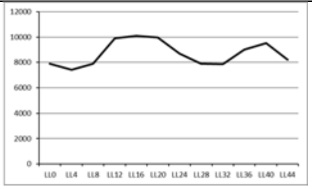

P53	catalytics;transferases;[acyl-carrier-protein] S-malonyltransferases ; binding	AT2G30200.1	32.00/41.52	4.30	20	32	66	52.90	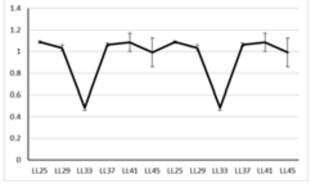	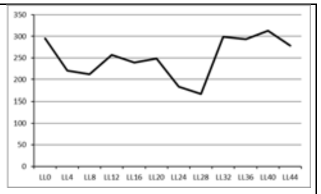
P54	U2A' : U2 small nuclear ribonucleoprotein A	AT1G09760.1	32.00/28.04	3.69	18	26	47	73.50	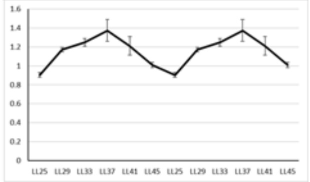	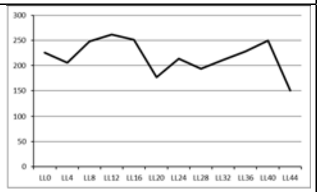
P55	30S ribosomal protein, putative	AT5G24490.1	32.00/34.85	5.37	12	18	67	36.00	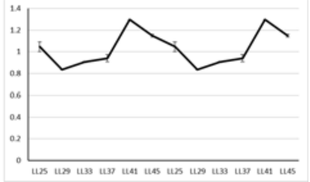	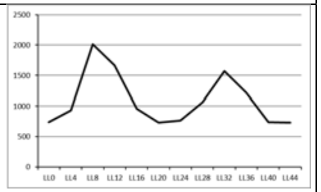
P56	GRF1, GF14 CHI : general regulatory factor 1	AT4G09000.1	32.00/29.93	4.16	17	25	49	67.80	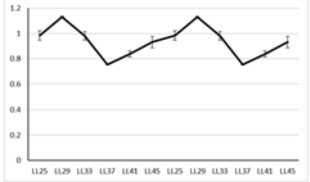	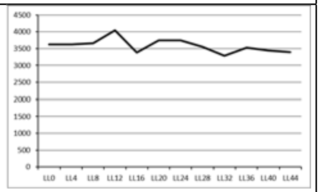
P57	THI1, TZ, THI4 : thiazole biosynthetic enzyme, chloroplast (ARA6) (THI1) (THI4)	AT5G54770.1	31.00/36.66	8.79	15	24	137	56.70	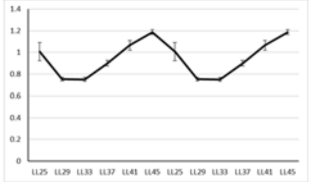	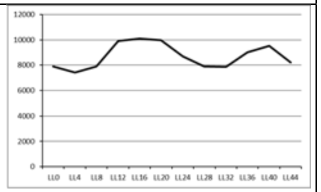
P58	CA1, ATBCA1, SABP3, ATSABP3 : carbonic anhydrase 1	AT3G01500.1	26.00/37.47	5.97	21	32	82	61.40	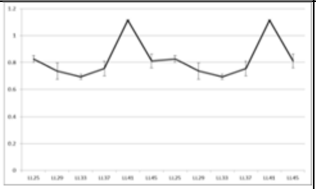	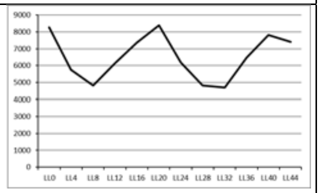

P59	ATGSTU1, GST19, GSTU1 | glutathione S-transferase TAU 1	AT2G29490.1	25.00/25.91	3.38	17	21	38	59.80	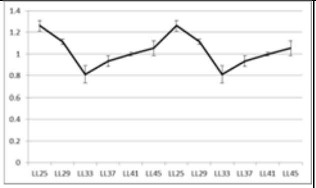	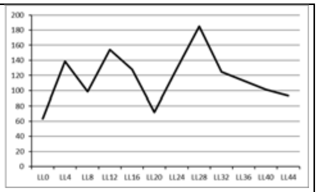
P61	lactoylglutathione lyase family protein / glyoxalase I family protein	AT1G08110.1	21.00/20.84	4.53	13	17	48	67.60	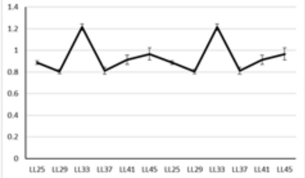	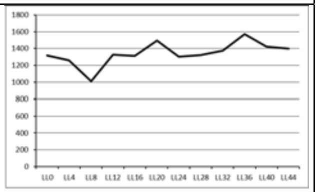
P62	Adenine nucleotide alpha hydrolases-like superfamily protein	AT3G11930.1	21.00/21.45	4.50	11	16	56	43.20	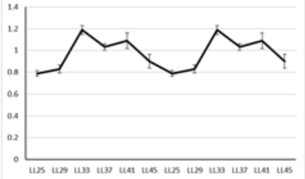	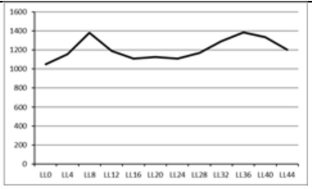
P63	ATPD | ATP synthase delta-subunit gene	AT4G09650.1	19.00/25.66	5.34	17	24	63	66.20	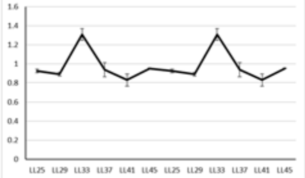	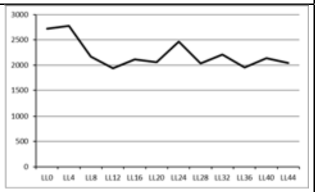
P64	ATPQ : ATP synthase D chain, mitochondrial	AT3G52300.1	18.00/19.58	10.30	28	37	100	89.90	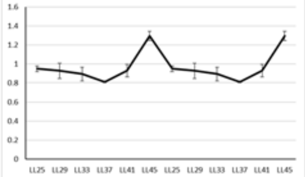	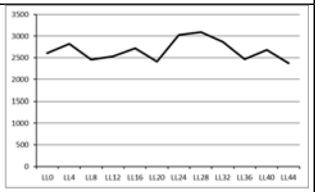
P65	ROC4 | rotamase CYP 4	AT3G62030.1<br>AT3G62030.2	17.00/28.20	3.09	8	12	37	36.90	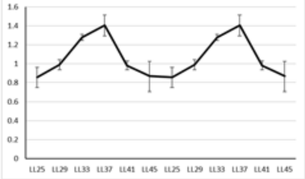	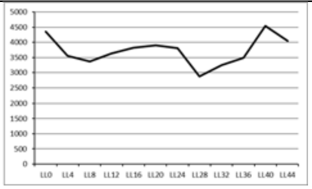

P66	SPF, MECPS : isoprenoid F	AT1G63970.1	17.00/24.81	2.08	6	8	17	35.50	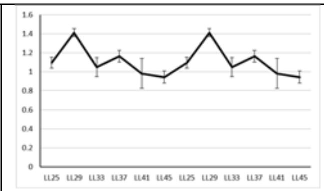	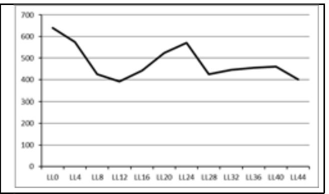
P67	Transcriptional coactivator/pterin dehydratase	AT5G51110.1	17.00/23.89	4.51	10	18	38	47.70	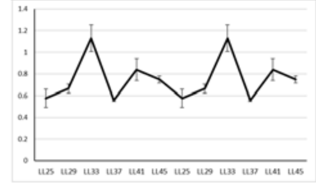	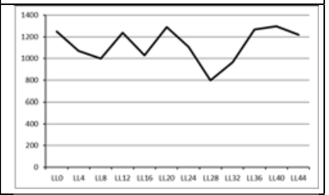
P68	FTRA2 : ferredoxin/thioredoxin reductase subunit A (variable subunit) 2	AT5G08410.1	16.00/20.14	9.15	9	12	80	38.00	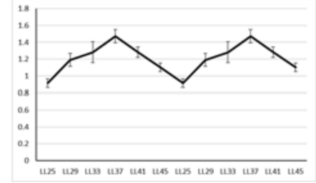	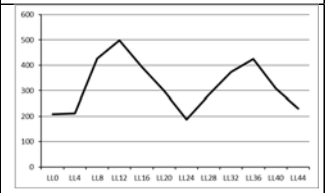
P69	ATGRP7, CCR2, GR-RBP7, GRP7 : cold, circadian rhythm, and rna binding 2	AT2G21660.1	16.00/16.89	7.67	18	22	63	84.10	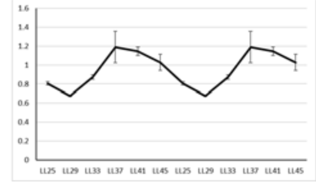	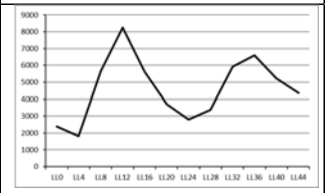
P70	Polyketide cyclase/dehydrase and lipid transport superfamily protein	AT4G23670.1	15.00/17.51	2.63	9	11	23	43.00	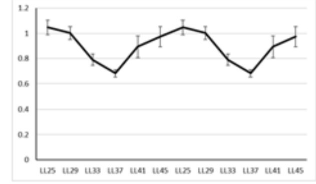	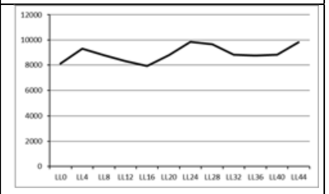
P71	Polyketide cyclase/dehydrase and lipid transport superfamily protein	AT4G23670.1	15.00/17.51	3.87	14	19	50	56.30	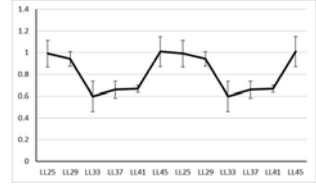	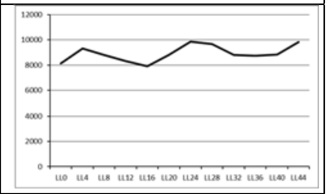

P72	ferredoxin thioredoxin reductase catalytic beta chain family protein	AT2G04700.1	15.00/16.43	2.33	10	13	23	63.70	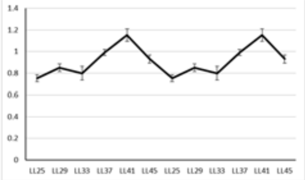	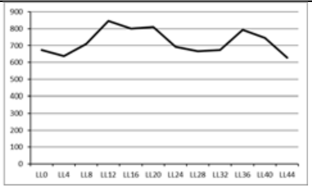
P73	RBCS1A : ribulose bisphosphate carboxylase small chain 1A	AT1G67090.1	14.00/20.21	1.45	8	8	13	46.70	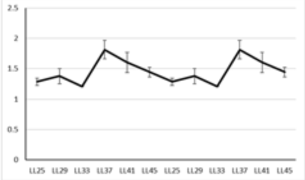	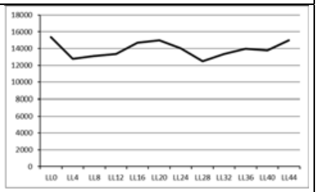
P75	endoribonuclease L-PSP family protein	AT3G20390.1	13.00/19.81	4.95	12	16	50	65.80	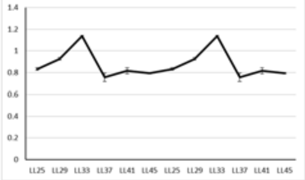	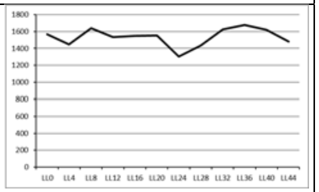
P76	TRXF1, ATF1 : thioredoxin F-type 1	AT3G02730.1	12.00/19.32	1.67	5	7	14	29.20	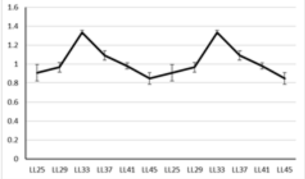	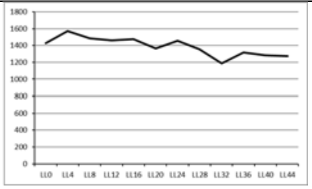
P77	Thylakoid soluble phosphoprotein TSP9 (InterPro:IPR021584);	AT3G47070.1	12.00/10.53	0.89	3	3	6	26.00	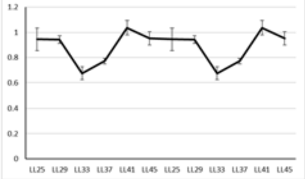	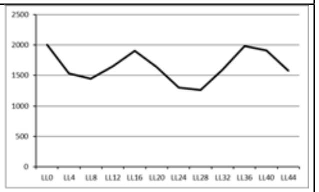
P77	ATRPB13.6, NRPB11, NRPD11, NRPE11 | DNA-directed RNA polymerase, RBP11-like	AT3G52090.1	12.00/13.56	0.89	2	3	6	23.30	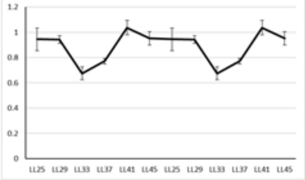	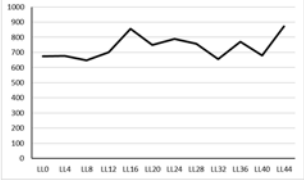

Notes:

1. Spot number as indicated on the 2D gel reference image; numerals indicate the spot numbers.
2. Protein identified through mass spectrometry.

3. Gene locus of identified proteins.
4. Theoretical molecular weight and experimental molecular weight.
5. Expression profile of proteins quantified through 2D gel analysis and identified by mass spectrometry across the time series under constant light. The data is double plotted to emphasize the oscillations. Y-axis values are protein expression profile shown as the average change in spot density at each time point. Error bars are the SD from 4 biological trials.
6. Transcript expression profile of corresponding proteins are based on the Diurnal (Mockler et al.,2007) data set LL12_LDHH (7 day old seedlings, 120 µmolm^{-}2 s^{-1}, 22°C, agar and 3% sucrose Kay, Harmer). Note these data begin at ZT0 so relevant comparisons with protein data (LL25) begin with the second half of the mRNA time series (LL24). Y-axis values are as described at Diurnal calculated from the Robust Multi-array Average (RMA) expression values.
