# Supplementary material for: Circadian Profiling of the Arabidopsis Proteome Using 2D-DIGE
Source: Front Plant Sci. 2016 Jul 12;7:1007. doi: 10.3389/fpls.2016.01007 (PMC4940426; doi:10.3389/fpls.2016.01007)
Supplement: Supplementary file 4 [file Table4.PDF]

Table S4: Expression profiles of rhythmic proteins and corresponding mRNA under constant light (RuBisCO depletion method)

Sample No[1.]	Protein ID[2]	Gene locus[3]	Thr MW /Exp MW[4]	% of spectra	No. of unique peptides	No. of unique spectra	No. of total spectra	% Sequence coverage	Protein expression profile[5]	Transcript profile[6] (Diurnal)
S01	CLPC, ATHSP93-V, HSP93-V, DCA1, CLPC1 | CLPC homologue 1	AT5G50920.1	103.50/103.45	5.04	18	19	43	23.30	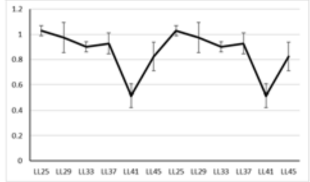	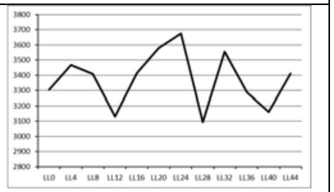
S02	CLPC, ATHSP93-V, HSP93-V, DCA1, CLPC1 | CLPC homologue 1	AT5G50920.1	90.035/103.45	12.80	50	62	148	49.30	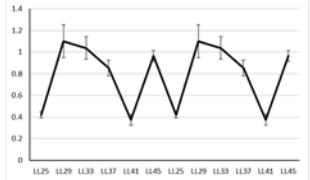	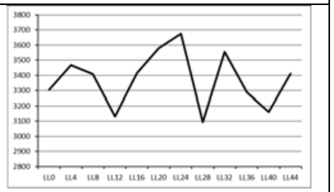
S03	ATSCO1, ATSCO1/CPEF-G, SCO1 | Translation elongation factor EFG/EF2 protein	AT1G62750.1	90.00/86.05	11	38	51	154	57.00	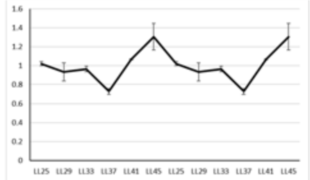	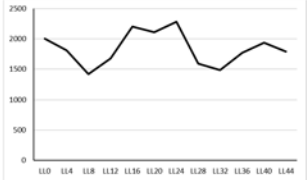
S06	PYK10, PSR3.1, BGLU23, LEB | Glycosyl hydrolase superfamily protein	AT3G09260.1	60.00/59.72	4.48	12	16	48	25.00	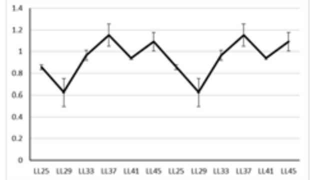	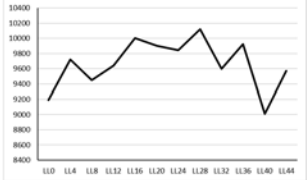
S07	PYK10, PSR3.1, BGLU23, LEB | Glycosyl hydrolase superfamily protein	AT3G09260.1	59.50/59.72	11.6	23	27	123	51.10	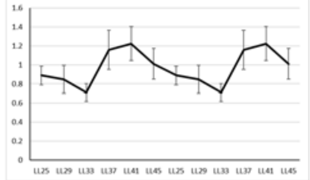	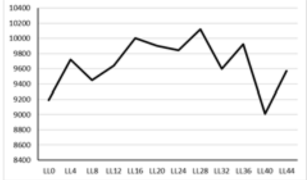

S08	ATTCP-1, TCP-1 | T-complex protein 1 alpha subunit	AT3G20050.1	59.40/59.23	6.53	32	40	99	66.10	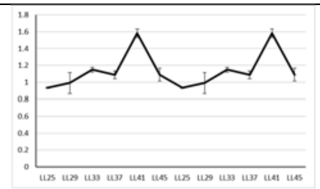	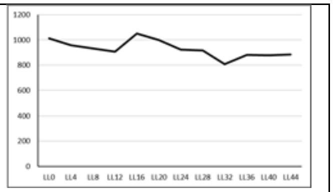
S09	TCP-1/cpn60 chaperonin family protein	AT3G11830.1	59.39/59.77	5.76	35	44	87	66.40	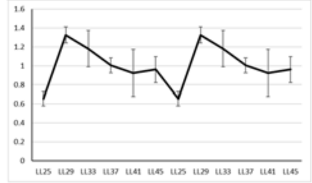	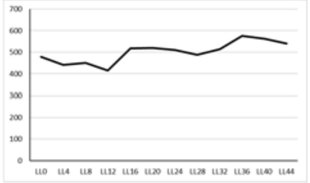
S10	KASI, KAS1 | 3-ketoacyl-acyl carrier protein synthase	AT5G46290.1	48.00/50.41	5.90	23	30	78	57.70	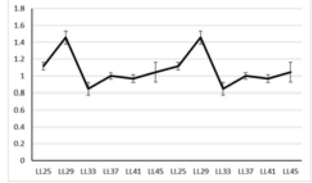	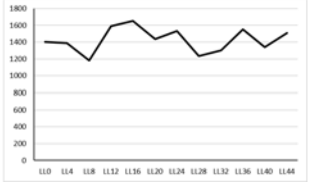
S11	RCA | rubisco activase	AT2G39730.1	47.00/51.98	15.20	29	44	216	61.40	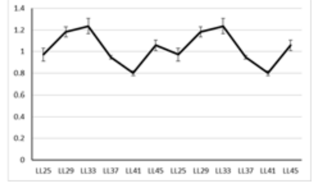	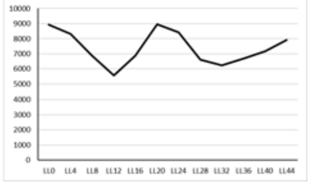
S13	ATPC1 | ATPase, F1 complex, gamma subunit protein	AT4G04640.1	41.00/40.91	7.47	16	19	110	42.90	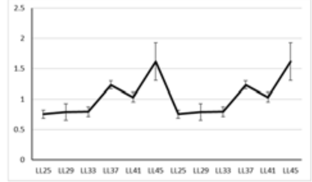	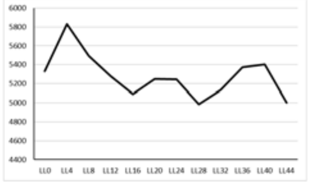
S15	GDSL-like Lipase / Acylhydrolase superfamily protein	AT1G29660.1	41.20/40.14	3.69	9	12	31	37.40	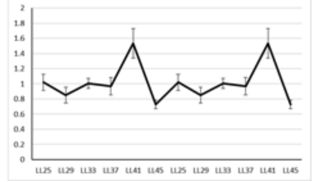	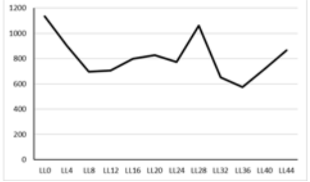

S15	SPDS1 | spermidine synthase 1	AT1G23820.1	41.20/40.67	3.69	9	13	31	29.90	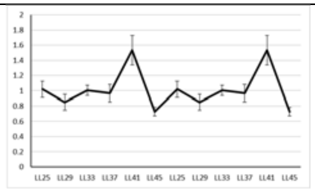	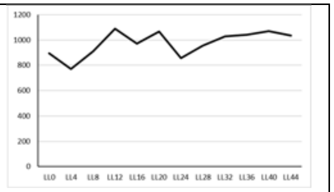
S16	NAD(P)-linked oxidoreductase superfamily protein	AT2G21250.1	38.00/35.01	4.81	22	26	67	54.00	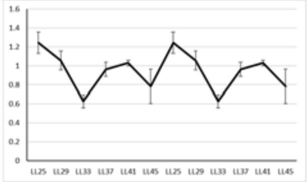	No data
S17	OTC | ornithine carbamoyltransferase	AT1G75330.1	41.00/41.00	3.38	10	14	48	25.30	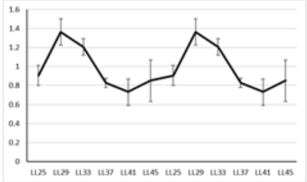	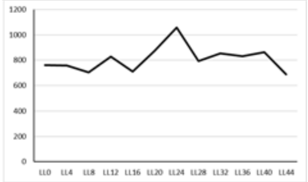
S18	LPA3 | Low PSII Accumulation 3	AT1G73060.1	45.00/40.03	2.25	12	13	24	40.80	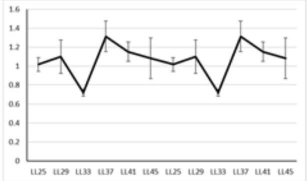	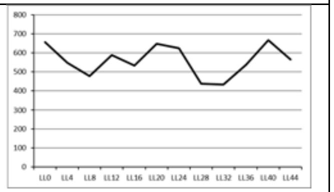
S22	PSBO2, PSBO-2, OEC33 | photosystem II subunit O-2	AT3G50820.1	35.00/35.01	3.94	14	20	51	48.00	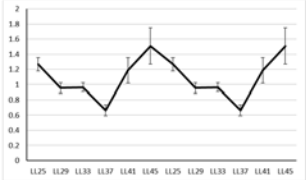	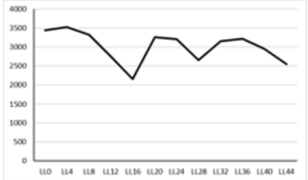
S23	PSBO2, PSBO-2, OEC33 | photosystem II subunit O-2	AT3G50820.1	34.00/35.01	5.39	15	22	72	47.40	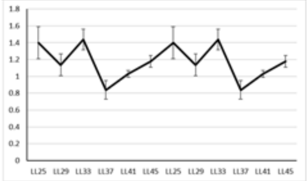	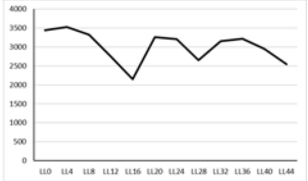

S25	PSBO2, PSBO-2, OEC33 | photosystem II subunit O-2	AT3G50820.1	34.50/35.01	7.34	27	37	111	68.00	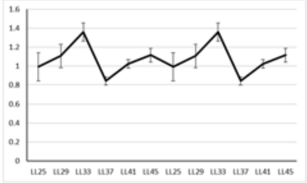	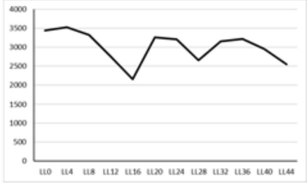
S26	TUF, emb2448, TUFF, VHA-E1 | vacuolar ATP synthase subunit E1	AT4G11150.1	31.00/26.06	3.42	14	16	39	60.90	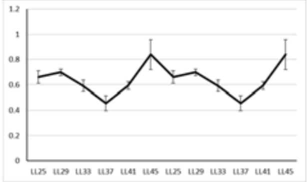	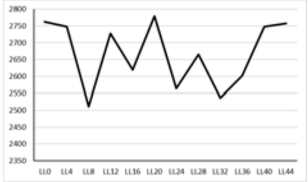
S27	ATCTIMC, TPI, CYTOTPI | triosephosphate isomerase	AT3G55440.1	30.03/27.16	4.93	17	25	62	66.90	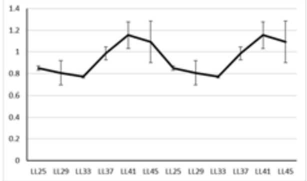	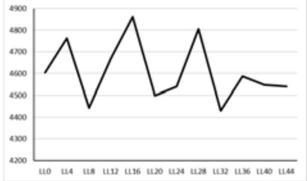
S28	CA1, ATBCA1, SABP3, ATSABP3 | carbonic anhydrase 1	AT3G01500.1	28.50/28.34	3.31	8	10	44	31.30	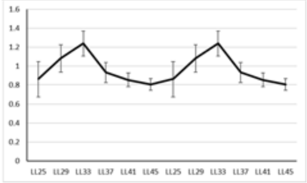	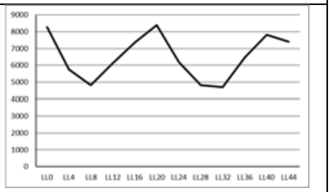
S30	ATGSTF8, ATGSTF5, GST6, GSTF8 | glutathione S-transferase phi 8	AT2G47730.1	28.00/29.23	2.97	15	21	42	48.70	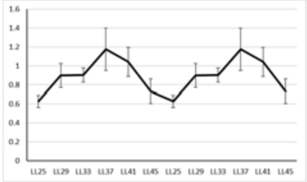	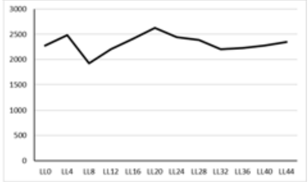
S31	ATGSTF8, ATGSTF5, GST6, GSTF8 | glutathione S-transferase phi 8	AT2G47730.1	27.00/29.23	2.61	12	16	34	42.20	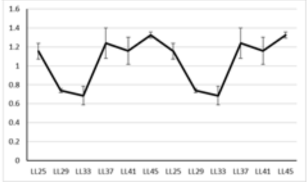	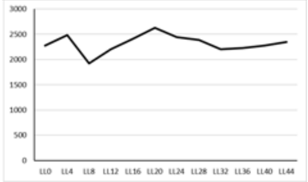

S33	MSD1, MEE33, ATMSD1 | manganese superoxide dismutase 1	AT3G10920.1	27.90/25.44	2.71	9	13	32	35.50	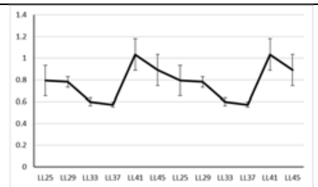	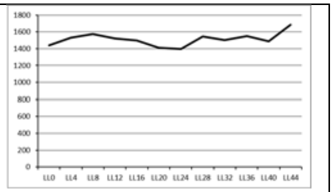
S34	FSD1, ATFSD1 | Fe superoxide dismutase 1	AT4G25100.1	25.04/23.79	2.20	6	8	26	21.20	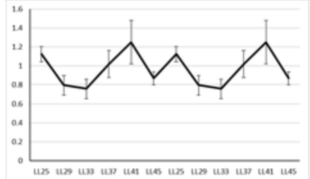	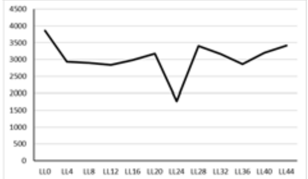
S35	Thioredoxin superfamily protein	AT5G65840.1	27.70/29.88	2.34	13	14	30	38.50	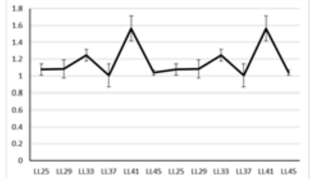	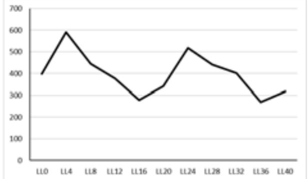
S37	PYR1, RCAR11 | Polyketide cyclase/dehydrase and lipid transport superfamily protein	AT4G17870.1	26.20/21.57	4.70	15	19	60	56.00	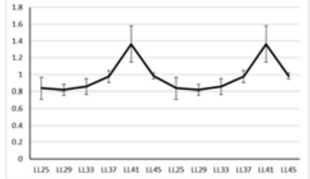	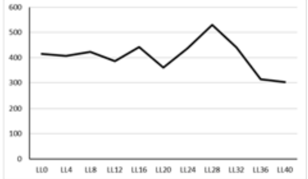
S39	CCH | copper chaperone	AT3G56240.1	25.00/12.96	3.44	10	13	37	72.70	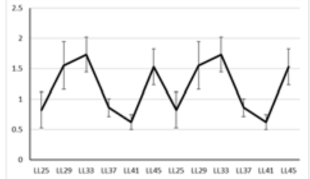	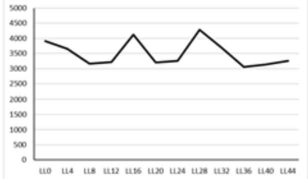
S42	PETC, PGR1 | photosynthetic electron transfer C	AT4G03280.1	24.45/24.36	2.39	7	9	22	2.39	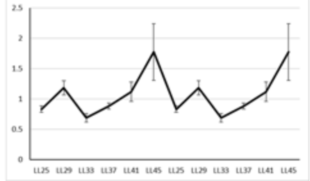	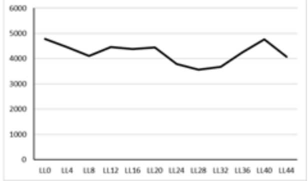

S44	Double Clp-N motif protein	AT4G12060.1	20.40/26.56	5.57	11	14	48	43.60	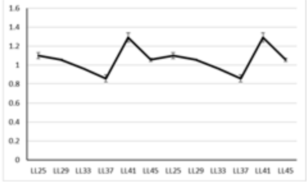	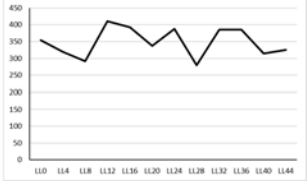
S45	Polyketide cyclase/dehydrase and lipid transport superfamily protein	AT4G23670.1	16.0/17.51	8.23	13	19	80	64.90	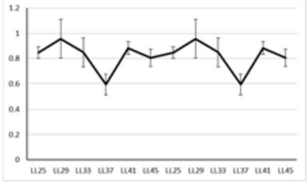	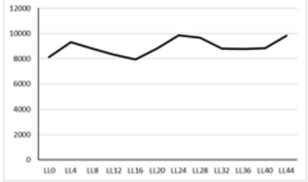
S47	PQL1, PQL2 | PsbQ-like 1	AT3G01440.1	13.00/24.78	3.99	10	12	26	44.10	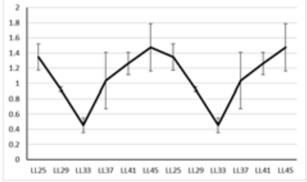	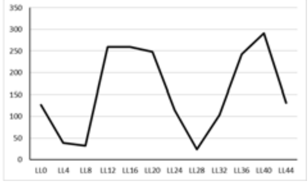
S49	Ribulose bisphosphate carboxylase (small chain) family protein	AT5G38410.1<br>AT5G38420.1	13.5/20.35	3.16	7	8	15	40.30	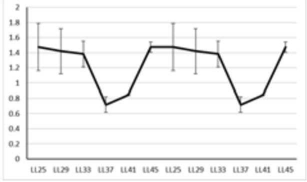	No data
S50	PGK1 | phosphoglycerate kinase 1	AT3G12780.1	48.5/50.11	5.28	30	42	83	69.20	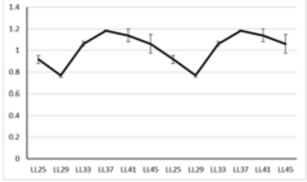	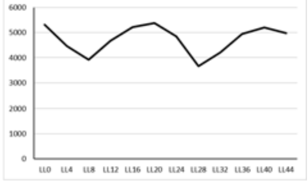
S51	Ribosomal protein L10 family protein	AT3G09200.1	37.90/34.13	6.04	15	21	76	41.90	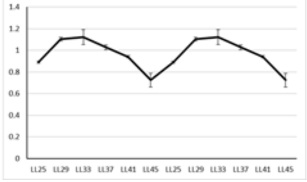	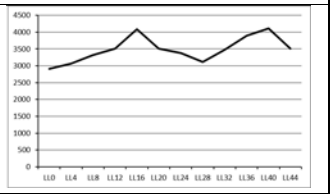

S52	GAPC, GAPC-1, GAPC1 | glyceraldehyde-3-phosphate dehydrogenase C subunit 1	AT3G04120.1	49.80/36.91	3.73	23	30	60	67.50	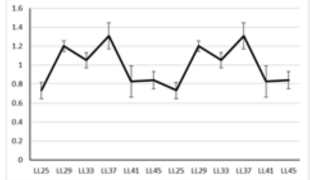	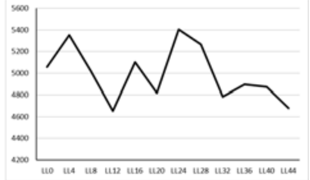
S54	endoribonuclease L-PSP family protein	AT3G20390.1	13.90/19.81	3.38	10	12	31	55.60	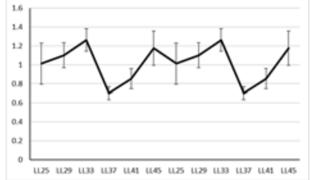	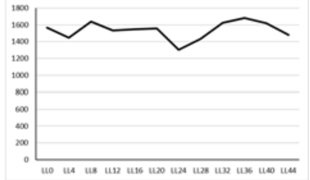
S56	ATPDIL1-1, ATPDI5, PDI5, PDIL1-1 | PDI-like 1-1	AT1G21750.1	62.50/55.60	5.36	26	33	64	49.50	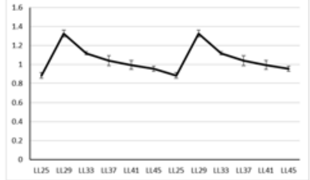	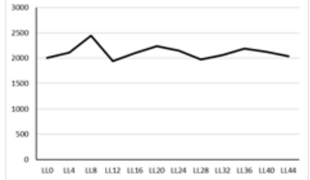
S58	ATPCAP1, PCAP1 | plasma-membrane associated cation-binding protein 1	AT4G20260.1	37.90/24.58	7.98	31	44	100	79.10	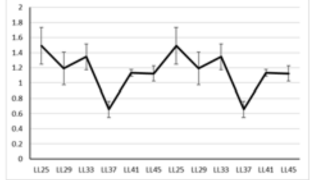	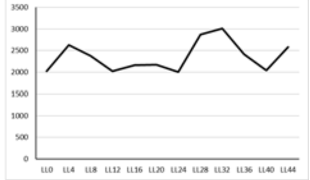
S59	ATGRP7, CCR2, GR-RBP7, GRP7 | cold, circadian rhythm, and rna binding 2	AT2G21660.1	16.90/16.89	4.10	13	14	31	83.00	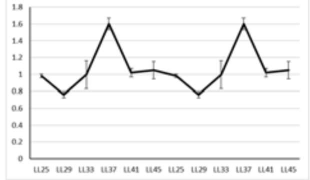	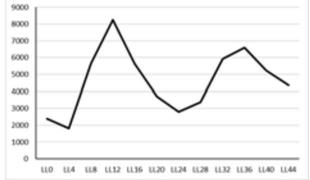

Notes:

1. Spot number as given on the 2D gel reference image, the numerals indicate the spot numbers.
2. Protein identified through mass spectrometry.
3. Gene locus of identified proteins.
4. Theoretical molecular weight and experimental molecular weight.

5. Expression profile of proteins quantified through 2D gel analysis and identified by mass spectrometry across the time series under constant light. The data is double plotted to emphasize the oscillations. Y-axis values are protein expression profile shown as the average change in spot density at each time point. Error bars are the SD from 4 biological trials.
6. Transcript expression profile of corresponding proteins are based on the Diurnal (Mockler et al., 2007) data set LL12_LDHH (7 day old seedlings, 120 µmolm^{-}2 s^{-1}, 22°C , agar and 3% sucrose Kay, Harmer). Note these data begin at ZT0 so relevant comparisons with protein data (LL25) begin with the second half of the mRNA time series (LL24). Y-axis values are as described at Diurnal calculated from the Robust Multi-array Average (RMA) expression values.
