# Supplementary figures and images for: Circadian Profiling of the Arabidopsis Proteome Using 2D-DIGE
Source: Front Plant Sci. 2016 Jul 12;7:1007. doi: 10.3389/fpls.2016.01007 (PMC4940426; doi:10.3389/fpls.2016.01007)

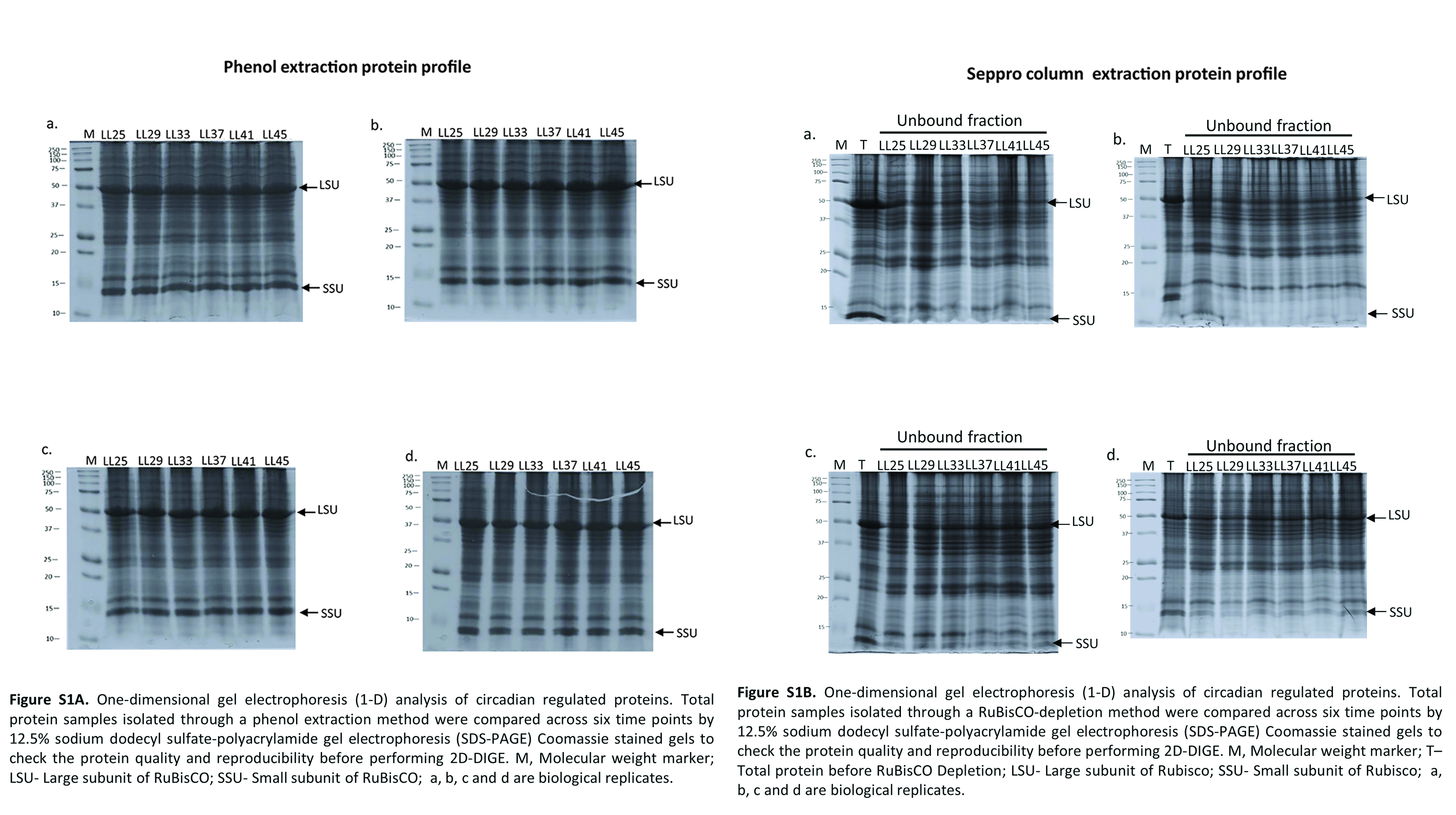

Supplement: Supplementary file 8 [file Image1.JPEG]

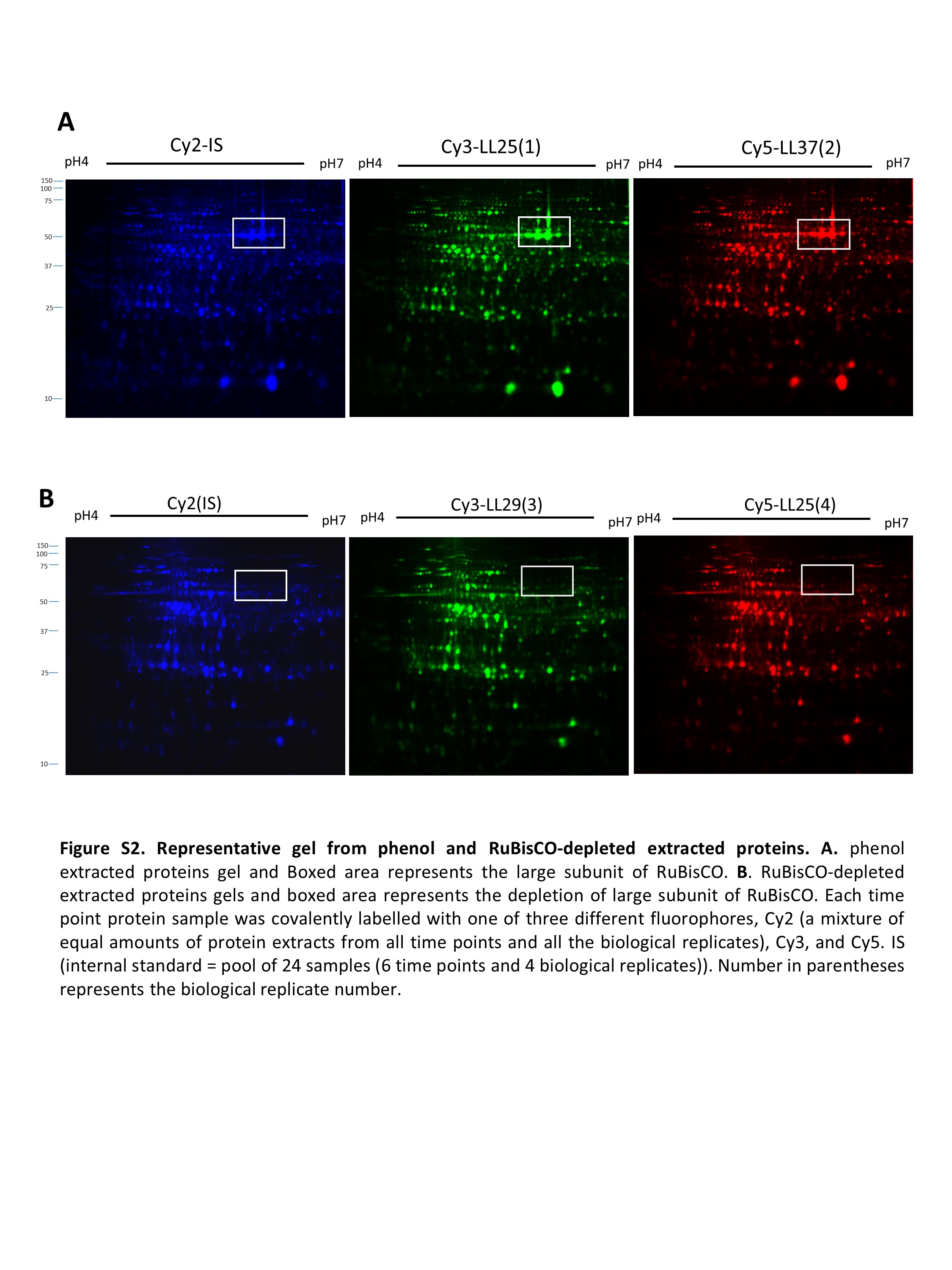

Supplement: Supplementary file 9 [file Image2.JPEG]

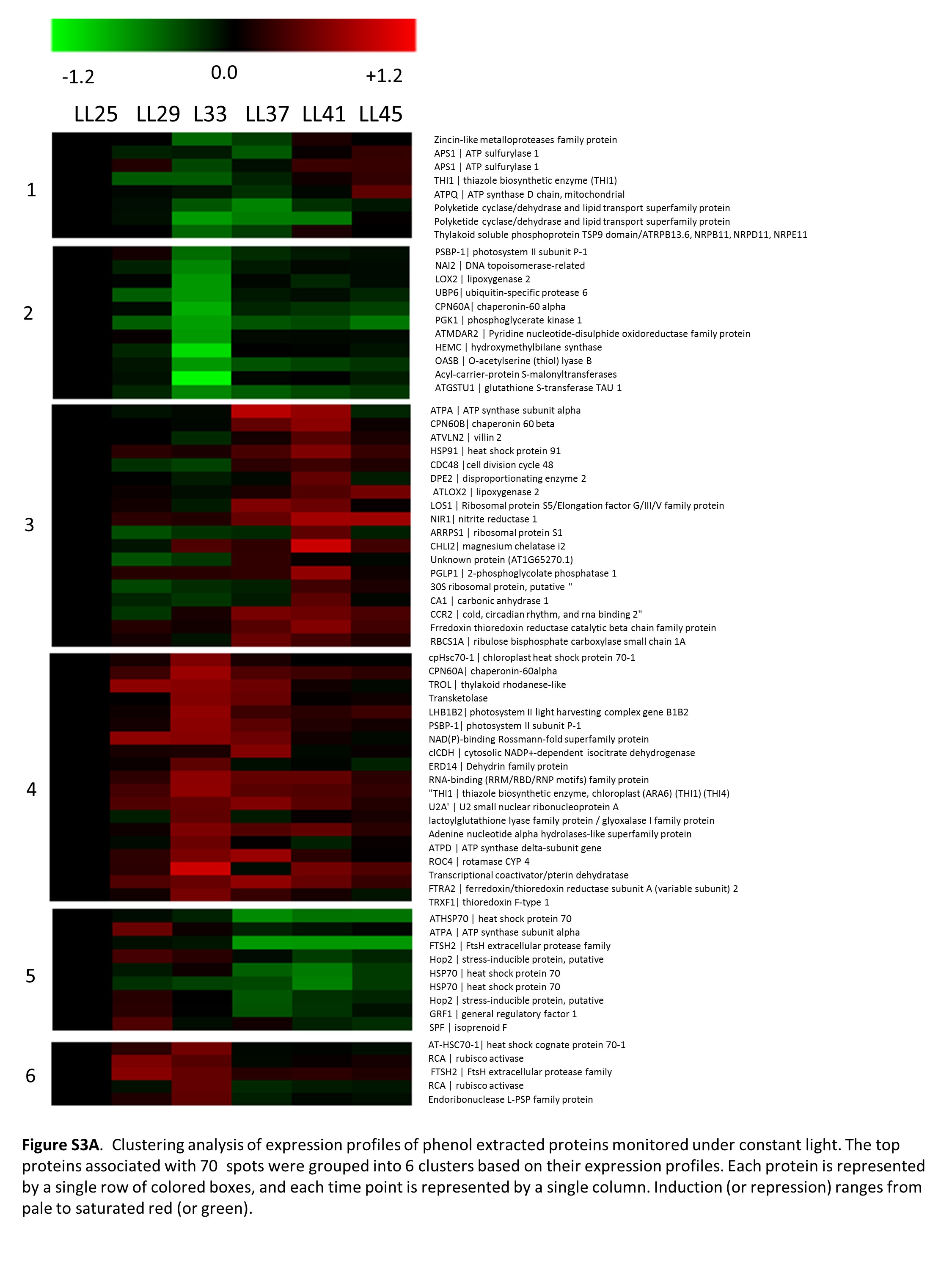

Supplement: Supplementary file 10 [file Image3.JPEG]

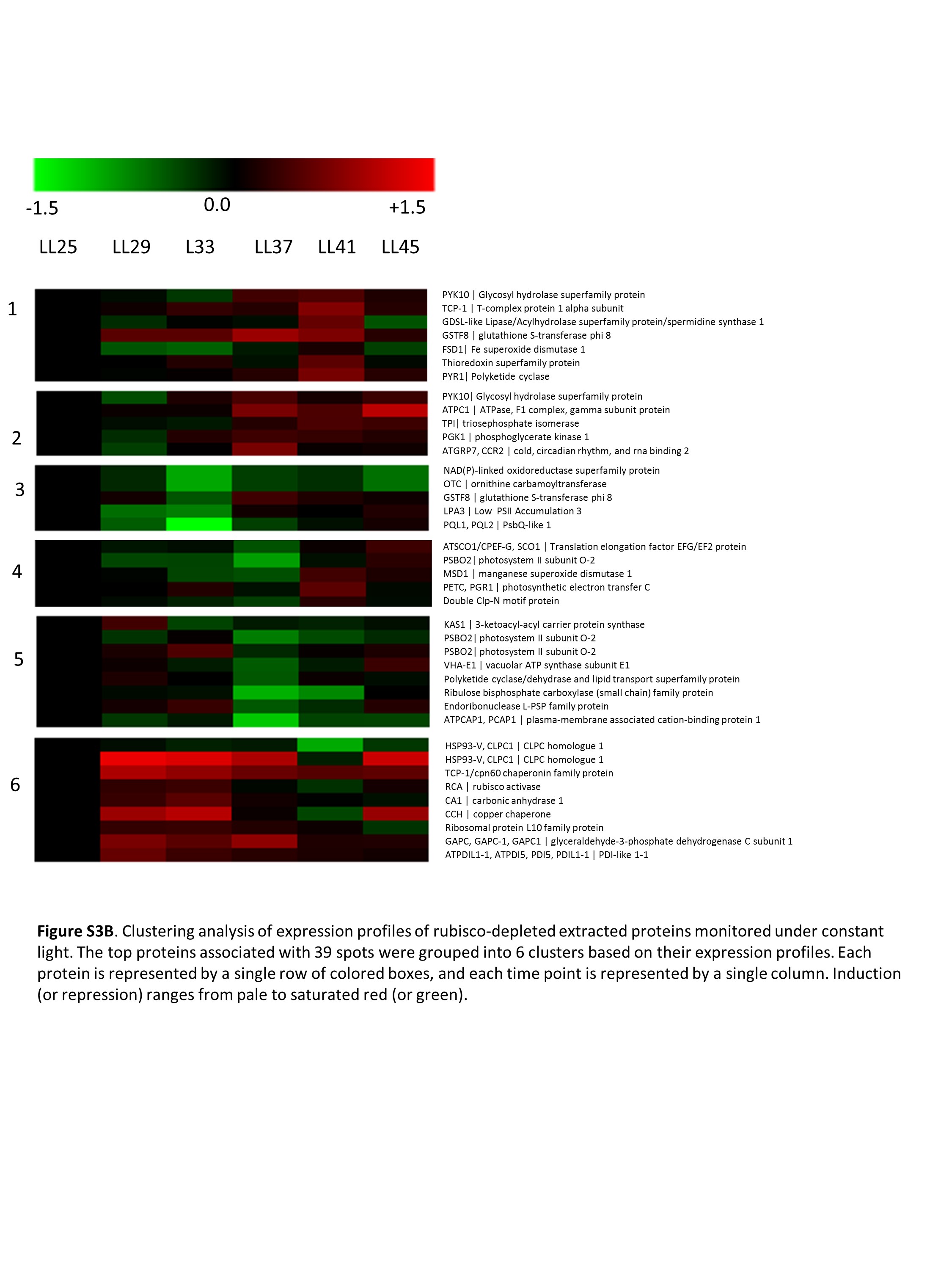

Supplement: Supplementary file 11 [file Image4.JPEG]

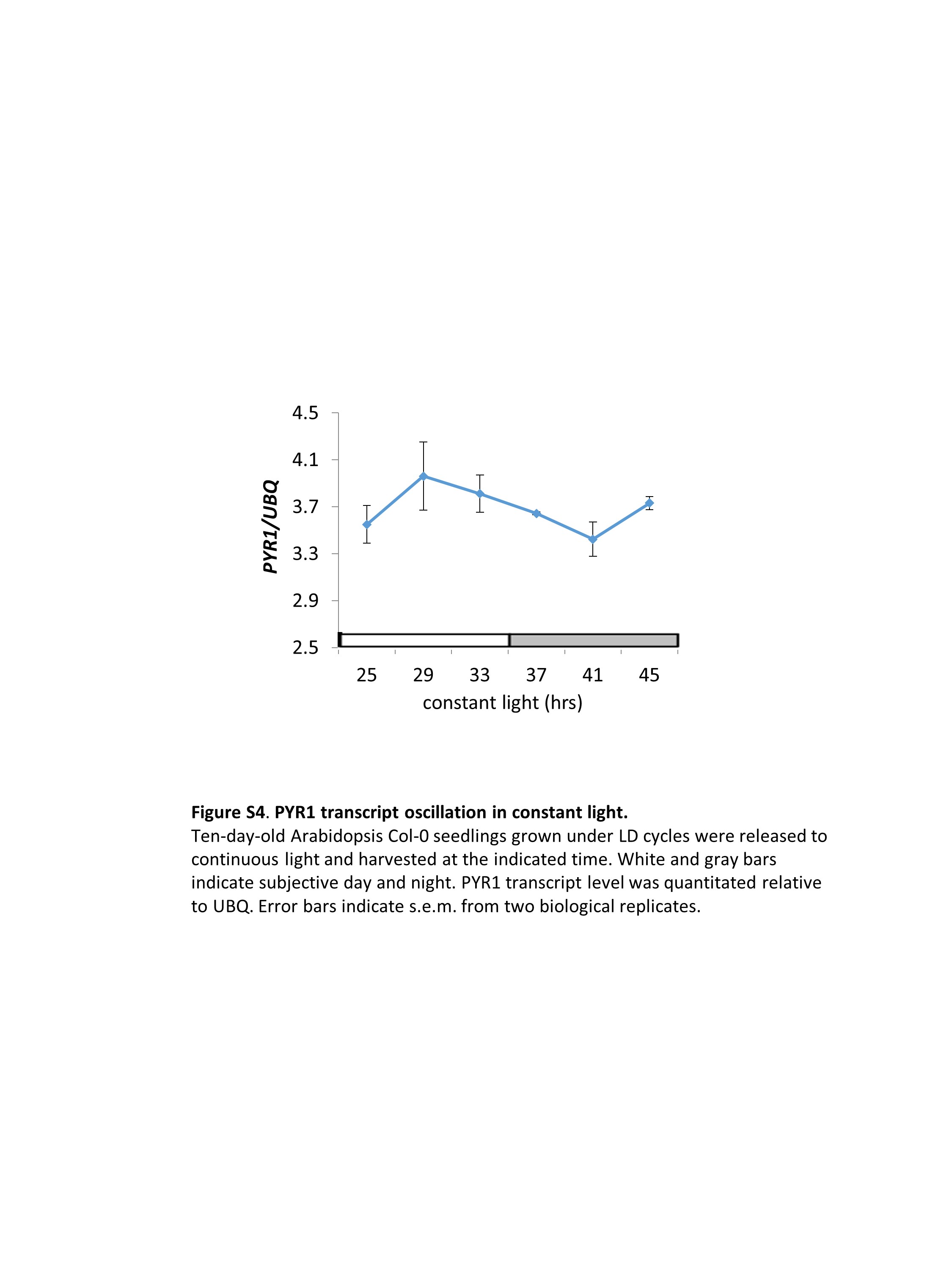

Supplement: Supplementary file 12 [file Image5.JPEG]
